# Technological advancements in the use of ionic liquid- membrane systems for CO_2_ capture from biogas/flue gas - A review

**DOI:** 10.1016/j.heliyon.2022.e12233

**Published:** 2022-12-15

**Authors:** Samuel Eshorame Sanni, Denen Ashiekaa Vershima, Emeka Emmanuel Okoro, Babalola Aisosa Oni

**Affiliations:** a Department of Chemical Engineering, Covenant University, Ota, Ogun, Nigeria; b Department of Petroleum Engineering, University of Port Harcourt, Choba, Rivers State, Nigeria

**Keywords:** Adsorption, Biogas, Biomass, Carbon capture, Membrane-ionic liquid system, Post-combustion

## Abstract

Carbon capture has become a very important method for curbing the problems associated with the release of carbon dioxide into the atmosphere, which in turn has detrimental effects on the planet and its inhabitants. Ionic liquids and membrane separation have been explored in this review paper as effective means of capturing carbon dioxide. An innovative approach to CO_2_ capture is the use of Ionic liquids (ILs) since they exhibit certain significant traits such as good stability (thermal, mechanical and chemical), inflammability and high absorptive capacities. Ionic liquids (ILs) are widely regarded as nontoxic substances. Viscosity and thermal degradation of ILs at temperatures slightly above 100 °C are the major disadvantages of ILs. Membrane separation is a technique used for the effective separation of substances by materials bearing holes in a continuous structure. Membrane technology has gained significant improvements, over the years. Several ILs and membrane systems were considered in this work. Their weaknesses, strengths, permeability, selectivity, operating conditions and carbon capture efficiencies, were all highlighted in order to gain a good perspective on ways by which the individual systems can be improved upon. The study considered several polymer-Ionic liquid hybrid materials as viable options for CO_2_ capture from a post-combustion process. Different ILs were scrutinized for possible integration in membranes by taking full advantage of their individual properties and harnessing their tune-able characteristics in order to improve the overall carbon capture performance of the system. Several options for improving the mechanical, chemical, and thermal stabilities of the hybrid systems were considered including the use of cellulose acetate membrane, nanoparticles (graphene oxide powder) alongside potential ionic liquids. Doping membranes with ILs and nanoparticulates such as graphene oxide serves as a potential method for enhancing the CO_2_ capture of membranes and this review provides several evidences that serve as proofs for this concept.

## Highlights

• Ionic liquid and membrane systems were considered as integral parts of a hybrid system.
• Prominent membrane and ionic liquids were considered based on their CO__2_ capture efficiencies_.
• A membrane-ionic liquid-graphene oxide system was proposed for capturing CO__2__ from flue gas.
• CO__2__ capture efficiencies >80% were recorded for the membrane-ionic liquid-graphene oxided system.

## 1 Introduction

### 1.1 Overview of carbon capture

The activities of humans have led to the rise in carbon emissions in the atmosphere and reports from literature have endeared researchers to believe that these activities are primarily responsible for climate change [1, 2]. In the last twenty years, there has been a relatively large increase in the average atmospheric temperature due to the high concentration of CO_2_ in the atmosphere. This has led to the increased interests from researchers and scientists on techniques and methods of addressing such issues [2, 3]. Some of the large-scale industrially used compounds from which CO_2_ is derived worldwide include urea (157 metric tonnes), salicylic acid (90 thousand tonnes), and cyclic carbonate (80 thousand tonnes) [4].

The task of trapping CO_2_ from the atmosphere is a difficult one since it requires the removal of an estimated 87 million tonnes of carbon dioxide every day in order to keep the CO_2_ levels from rising too high [5]. Several research works have been tailored towards capturing CO_2_ in the atmosphere, however, the most feasible means of capturing the gas is usually at the carbon emission source which includes power plants that consume large fossil fuels [6, 7, 8]. Carbon capture and storage (CCS) has sparked significant interests in the research space, which has led to a resultant reduction in the global CO_2_ emissions, less energy consumption and the substitution of fossil fuels with low/non–CO_2_–emitting alternative energy sources. Combining CCS with other greenhouse gas (GHG) reduction programs will lead to a significant cut in the cost of climate change mitigation significantly [9].

The practice of carbon capture has been exercised over a period of time however, efficient CO_2_ capture and storage techniques are still being developed owing to lack of available and efficient carbon capture systems. CCS, in relation to combustion chemistry, has been accomplished, however, the moderate progressive advances recorded so far have been due to the broad portfolio of hydrocarbon fuels that serve as major energy sources. The impact of greenhouse gases (GHG) has revealed to the world that a rise in the amount of carbon dioxide in the atmosphere, will lead to a gradual warming of the planet and the resultant effect will bring about dramatic/severe weather changes and poor air quality which may leave the earth in a state where it breeds new varieties of diseases that may be harmful to its inhabitants [10, 11].

The persistent challenges associated with climate change have led to the assemblage of 196 parties at the 21^st^ conference of parties (COP21) which held in Paris on the 12^th^ of December 2015 in a bid to endorse an international treaty on climate action. The aim is to reduce the global warming effect to degrees below 2 and 1.5 °C. In lieu of the Paris agreement, alternative energy sources have been explored in other to control carbon emissions in the atmosphere and due to lack of suitable options, natural gas still remains the only option that can help meet up the Paris agreement target (i.e., 100% reduction in the net global emissions by 2100) [12]. Options for producing renewable fuels from biomass have gained an alarming increase owing to the significant quantity of biomass, biogas and biochar released by the material after combustion.

Some literature dedicated to membranes and ionic liquids (ILs) for CO_2_ capture from biogas include: the work of Yusuf et al. [13] which involves the synthesis of a novel carbon-IL system for the capture of CO_2_ from biogas. The results showed a high CO_2_ adsorption capacity of 84.89 mg of CO_2_ per gram adsorbent. In addition, Cichowska-Kopczyńska et al. [14] synthesized different membrane-IL systems for CO_2_ capture from biogas. The results showed a high CO_2_ permeability of 1888 ± 117 barrer for polypropylene-[EMIM][Tf_2_N] at a temperature of 298 K. Friess et al. [15] synthesized a membrane-IL (Polytetrafluoroethylene-[EMIM][Tf_2_N]) system for CO_2_ capture from biogas. They obtained a high permeability of 525 barrer for 75 wt % of the IL. Despite the availability of literature for CO_2_ capture from flue gas using membrane-ionic liquid systems, there is a scarce volume of literature for CO_2_ capture from biogas using membrane-ionic liquid systems.

Biogas from biomass consists of 50–65 % CH_4_, 30–45 % CO_2_ and other trace gases like H_2_S, H_2_ and O_2_, while flue gas consists of 66% N_2_ from air and 33% CO; CO in the presence of excess oxygen is subsequently oxidized to give CO_2_ which poses challenges in process systems and the environment. Although its CO_2_ content is less than that of fossil fuel, biogas from biomass contributes significantly to the carbon footprint of the globe, which then informs the need for improved technologies to abate unforeseen consequences. Of the several and commendable capture technologies proposed in literature, none seems to have considered the measure of viability in taking advantage of some special/potential hybrid membrane-ionic liquid systems as a post combustion capture technology for CO_2_ capture; this then led to the motivation for this research where several ionic liquid and membrane systems were scrutinized with the intention of taking advantage of their inherent properties for potential application in CO_2_ capture from flue gas/biogas released after combustion. The strategic approach adopted here strives to open up new frontiers for the integration of novel membrane-ionic liquid systems in combustors for the efficient trapping of CO_2_ rather than releasing the gas into the atmosphere, which may in turn constitute environmental nuisance as well as pose health risks.

There are some available methods for capturing CO_2_ and some of them include, adsorption (membrane separation, the use of activated carbon, metal-organic frameworks (MOFs), and zeolite), absorption (the use of absorbents such as methanol amine, ionic liquids, and other solvents), chemical looping, and cryogenic separation [16, 17, 18]. Reduced energy intake, low maintenance costs, and applicability at various scales are all possible with solid sorbents. First, the necessity to construct a very big structure at a low cost while enabling the entire structure to be periodically isolated from air during the regeneration process within some specified temperature, pressure, or humidity is difficult for solid sorbent designs. Additionally, there are fundamental issues associated with incompatible requirements such as excellent sorbent performance, low cost, and long economic life under ambient conditions. The most commonly used method for CO_2_ capture is the absorption method and it is used by most industries universally at low-cost, because it does not require high energy utilization [6].

Ionic liquids (ILs) are a unique class of salts that are rapidly spurring academic interests [19]. Based on their unique physiochemical characteristics which include chemical/thermal stability, low vapor pressure, non-flammability, wide liquid state temperature range and favorable solubility, they are also known to be environmentally friendly chemical solvents for chemical processes [20, 21]. ILs can be designed for specific purposes by altering certain unique properties within them. Thermally, electrochemically, radiolytic, and chemically stable ILs are rare in literature. Almost all of the applications of ILs, which include desorption, catalysis, and solvent-regeneration, involve a temperature that is relatively high. The great thermal stability of modified ILs, or their resistance to evaporation and disintegration, is one of the main factors contributing to their recent popularity. The requirement for the widespread industrial use of modified ILs is thermal stability. There are two different types of thermal effects on pristine ILs: thermal degradation/decomposition and evaporation. The term "decomposition of ILs" refers to a chemical reaction that results in the creation of new compounds. The physical transition of ILs from a liquid to a gaseous state occurs without the creation of any new types of substances. Evaporation usually occurs along with IL breakdown. Contrarily, volatile ILs do not always result in IL breakdown, especially under conditions of low temperature and low pressure. Lu et al. [20] synthesized and characterized a novel IL known as diethanolamine glycinate ([DEA][GLY]) for CO_2_ capture. The measured CO_2_ solubility in the [DEA][GLY] IL was discovered to be significantly higher than that of classic ILs such as [bmim][BF_4_] IL. Yang et al. [22] conducted experiments in an absorption-desorption loop system in a bid to evaluate the effects of SO_2_ and O_2_ on CO_2_ capture using an aqueous amine solution mixed with IL. The CO_2_ removal efficiency of the aqueous amine solution combined with [bmim][BF_4_] was above 90 % and the SO_2_ concentration at the absorber outlet was less than 20 ppb (parts-per-billion). The high viscosity of ILs serves as a major disadvantage for utilizing pure ILs [23].

Membrane separation is a technique for effectively separating substances using pores and voids in a continuous polymer network, thus the feeding and exiting of filtered gas are possible in this system. During carbon sequestration and storage, the membrane adsorbs desirable components while permeating the unwanted components, thus resulting in gas mixtures being separated. Prior onward transportation and storage, CO_2_ must be isolated from exhaust gas streams [12, 24, 25]. Carbon has an atomic size of 0.15 nm and needs to be selectively separated from the other constituents of flue gas. By manipulating the pore sizes of membranes to fit the diameter of carbon molecules, researchers have found a way to selectively remove carbon from flue gas/biogas [4]. By selecting an appropriate pore size for the membrane-support, a hybrid ionic liquid system can withstand a reasonably high trans-membrane pressure. In general, membranes having pore sizes ranging from 100-200 nm are appropriate for creating hybrid ILMSs. The physiochemical properties of membranes, permeability of adsorbate/gas molecules and the differential partial pressures of the adsorbate all influence the overall sequestration of the gas by membranes. Chen and Ho [26] successfully adopted high-molecular-weight polyvinylamine (PVAm)/piperazine glycinate (PG) membranes for CO_2_/N_2_ separation from flue gas. To increase the degree of adhesion between the coating solution and the substrate, sodium dodectyl sulfate (SDS) was added as a surfactant to the coating-solution. Membranes with thicknesses ranging from 100 to 200 nm were coated on various substrates. At 57 °C, and in the confines of 17 % water vapor, the membrane had a CO_2_ permeance of up to 1100 gas permeation unit (GPU) and a CO_2_/N_2_ selectivity of more than 140; during a 20-hour stability test, the membranes were seen to have retained their stability.

Unfortunately, for most membranes, selectivity and permeability are two conflicting properties as extremely selective compounds have low permeability, whereas very permeable membranes are typically non-selective [27, 28]. Due to the recent advances made in the development of new techniques for CO_2_ capture, scientists and researchers have began considering combining membranes and ionic liquids. This approach takes full advantage of their individual strengths and synergistic properties as a way of compensating for their individual weaknesses. Solution-diffusion is the typical process used for gas transport through polymeric materials/membranes. The inherent adsorption or permeation of CO_2_, CH_4_, N_2_, and O_2_ informs the gases being treated/separated. Because CO_2_ has a higher critical temperature than N_2_ and O_2_ (which indicates that it is more condensable), the solubility and selectivity of CO_2_/N_2_ pair are always greater than those of CO_2_/O_2_ and CO_2_/CH_4_. Additionally, CO_2_ can interact with polar groups in the materials to increase its solubility because, it has a larger quadruple moment than N_2_ and O_2_ [29]. Additionally, compared to N_2_ and O_2_, CO_2_ has a smaller kinetic diameter (but a larger critical volume), therefore its diffusivity and selectivity increase as the materials' capacity for molecular-sieving increases.

**Figure 1:**
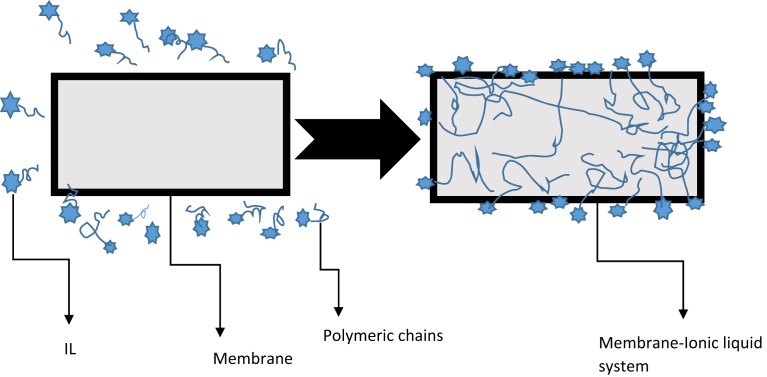
Mechanism for the formation of a membrane-ionic liquid system.

Figure 1 presents the distribution of ILs in polymeric chains. The ILs are mostly made up of dissociated cations, anions, and some ionic pairs. Ionic liquids or any other plasticizing agent can be added to a membrane to change the degree of crystallinity as well as the mobility of the polymer backbone which is partly what increases their ionic conductivity. In addition to offering mobile ions, it has been discovered that ionic liquids also result in increased amorphicity or decreased crystallinity of membranes [30]. Complexation of ionic liquid cations with a polymer backbone causes structural modification in the polymer, which in turn affects polymer-crystallinity.

The focus of this study is on the use of membrane-ionic liquid systems for capturing CO_2_ from a post-combustion process. Although various membrane systems and ionic liquids have been employed in trapping carbon dioxide, the combination of membranes and ionic liquids with variations in permeability and selectivity has not been widely explored. Hence, owing to the high prospects in the use of these combinations in trapping CO_2_, such systems are being projected from a unique point of view where various membrane and ionic liquid systems are considered in a bid to harness their relative strengths, weaknesses, inherent properties, deficiencies and synergistic effects for capturing CO_2_ from a post-combustion process.

### 1.2 Post-combustion technology

CO_2_ from fossil fuel-powered facilities is captured using three different strategies: oxy-, pre-, and post-combustion techniques. Post combustion remains the most widely sought-after technology for capturing CO_2_, because it can be easily adjusted to the system of most industries without completely altering/modifying their inherent unit operations [31].

Post combustion technology is associated with the capturing of CO_2_ from a combustor before it transits into the atmosphere and it is a prominent technology applied to power plants [32, 33]. It involves the removal of CO_2_ after the source-fuel is burnt [34]. The flue gas generated after the burning of the fossil fuel consists of CO_2_ of low partial pressure and less than 20% CO_2_-concentration. Despite the usual differences in CO_2_ concentrations generated during combustion, most of the industrial capture processes from cement industries, steel industries, power plants etc. that utilize fossil fuels in their routine processes, are largely post combustion-related [35]. The flue gas mixture comprises of several gaseous constituents including nitrogen, oxygen and water vapor which are fed into a post combustion carbon capture system that is attached downstream of the industrial plant [31].

The advantages and disadvantages associated with using a post-combustion carbon-capture system are as contained in Table 1.

**Table 1:** Advantages and disadvantages of a post-combustion system.

Advantages	Disadvantages
• Environmental CO_2_ emission reduction	• Researchers and scientists anticipate that post-combustion carbon capture devices will increase the cost of electricity by 70 %.
• Compared to other technologies, much more research has gone into its study and application	• The effect of low CO_2_ concentration on unit capture efficiency in coal-fired and gas-fired facilities is a considerable disadvantage.
• The recovered CO_2_ can be pumped into depleted layers containing oil and gas reservoirs. The pressure inside the reservoirs is increased by gas injection, which provides the driving force that makes it very easy to extract the oil and gas, while CO_2_ is permanently deposited underground.	• CO_2_ transportation pipelines can cause accidents.
• Extremely adaptable	• As a result of the high cost of building and maintaining a post-combustion carbon capture system, most businesses are not interested in this technology.
• Fertilizers such as ammonium sulfate and ammonium nitrate can be obtained via the ammonia absorption process.	• Because of its toxicity, using ammonia solution in post-combustion absorption processes might lead to severe consequences on the environment.

### 1.3 Membrane-ionic liquid systems

Incorporating liquids and solid reinforcements such as nanoparticles in polymeric materials is one technique for enhancing the permeability of gas adsorbents while retaining their selectivity [36]. Therefore, it is critical to choose micropore diameter supports that permit the liquid to be retained in the pore by capillary force in order to ensure the long-term durability of ILs in the pores of the polymer [37]. In recent years, ILs paired with membrane systems have emerged as potential separation systems [38]. The membrane-support can help the paired IL retain its solvent characteristics, thus improving gas separation efficiency [39]. Low performance of membrane-IL systems may occur as a result of the configuration of the membrane pores, as well as high temperature and pressure. Membrane-IL systems cannot be used in high temperature operations as the process may cause several damages to the system, thus rendering it ineffective. This problem can be solved by incorporating nanoparticles such as graphene oxide powder, which helps to improve the mechanical strength of the material. Several distinct types of membrane technologies integrated with ILs have been studied in recent years and these include supported ionic liquid membranes (SILMs), IL composite polymer membranes (ILPMs), ionic liquid composite mixed matrix membranes (ILMMMs), poly(ionic liquid) membranes (PILMs), ionic liquid gel membranes (ILGMs) and ionic liquid membrane contactors (ILMCs); these systems were found to exhibit high gas separation tendencies.

#### 1.3.1 Supported ionic liquid membranes (SILMs)

Supported ionic liquid membranes (SILMs) are systems that integrate membranes with ionic liquids (ILs) for efficient gas separation [40, 41]. Gas penetration across supported ionic liquid membranes (SILMs) has been reported in a number of publications [41, 42]. The majority of these research efforts were focused on pure gas analyses involving carbon dioxide (CO_2_), Nitrogen (N_2_), Hydrogen (H_2_), and methane (CH_4_) [42,43]. Most of the investigations have been conducted on alkyl methylimidazolium cations and several anions including those of tetrafluoroborate (BF_4_) [38]. It is widely acknowledged that the anion of an IL has a stronger influence on gas permeability and selectivity than its cation [42, 43, 44]. Grünauer et al. [43] synthesized three distinct membrane-ionic liquids for CO_2_ capture. The common anion for the different ionic liquid cations (1-benzyl-2-methylpyridinium [Bz_2_Py], benzylpyridinium [BzPy], 1-benzyl-4-methylpyridinium [Bz_4_Py] and 1-benzyl-3-methylpyridinium [Bz_3_Py]) was bis(trifluoromethylsulfonyl)imide [Tf_2_N], while CO_2_/N_2_ separation was achieved at 298 K and 0.35 bar with polytetrafluoroethylene polymer (PTFE) membrane as support. The highest CO_2_ permeability (518 barrer) was recorded for [BzPy][NTf_2_], while the highest selectivity (33.1) was recorded for [Bz_2_Py][NTf_2_]. Fan et al. [40] synthesized (2-hydroxyethyl)-trimethyl-ammonium(S)-2-pyrrolidinecarboxylic ([Choline][Pro]) mixed with polyethylene glycol (PEG) 200 as support for polyethersulfone (PES) membrane for CO_2_ capture from a mixture of CO_2_ and N_2_. The experiment was carried out at a temperature of 308 K and a pressure of 1.8 bar. The selectivity and permeability of CO_2_ were 34.8 and 343.3 barrer, respectively.

#### 1.3.2 IL polymer membrane (ILPM) composites

Researchers all across the world are making concerted efforts to create new membrane materials that can effectively remove CO_2_ from a mixture of several light gases including nitrogen (N_2_) [45,46]. Polymers are one of the most commonly investigated membrane materials for trapping carbon dioxide due to the ease with which they are processed as well as their low costs of procurement [45]. However, Robeson allotted the limitation of polymers for CO_2_ capture to the increase in permeability which leads to a decrease in selectivity, as depicted in upper bound curves, which they confirmed is one of the major shortcomings of membrane adoption in gas separation technologies [46]. As a result, a large number of researchers are working on developing novel membrane materials or modifying the existing ones in order to boost gas separation efficiency [47].

Polymer blending is thought to be an appealing technique for optimizing the inherent properties of membrane materials which helps to increase their mechanical strengths and gas separation tendencies [48]. Polymer blending, on the other hand, is straightforward, repeatable, and feasible when compared to polymer-blocking or cross-linking.

**Figure 2:**
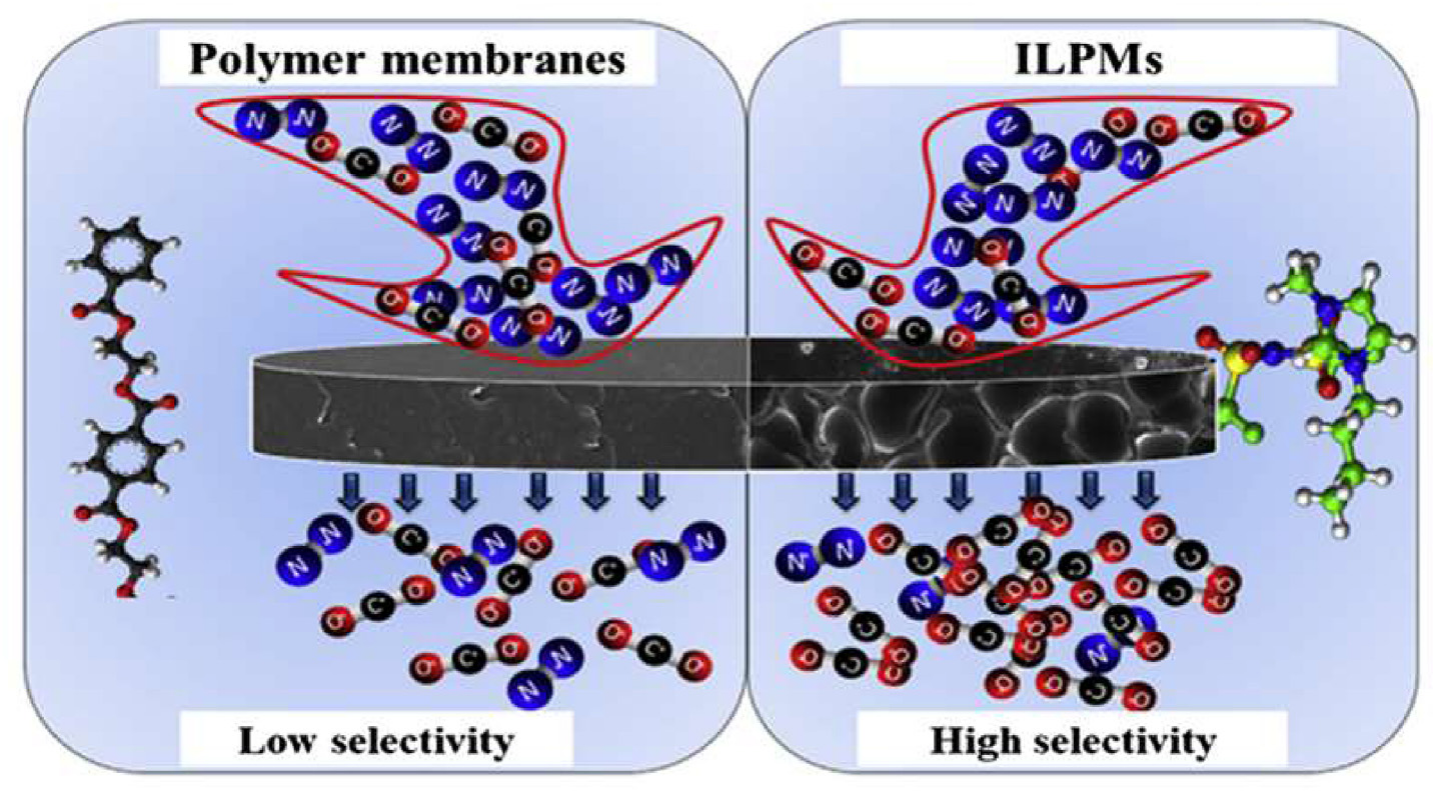
Structure of ILPMs. Adopted from Yan et al. [49].

ILPMs are made of clusters of materials that improve the mechanical stability of membranes (Figure 2). As previously stated, SILMs have been proposed as suitable options for modifying polymers. In terms of operating pressures, SILMs are limited. SILMs are often confronted with serious failure problems/performance inefficiency, especially when the pressure differential across the membrane is significant enough to overcome the interaction between the inter-layer and the liquid solvent [48]. Thus, the liquid inter-connectivity through the pore-support is pushed away, hence causing the membrane to no longer function as a barrier with significant performance-drop [50]. Integrating ILs into a polymeric material can be a viable strategy for overcoming SILM limitations [51].

The development of polymer/IL composite materials, where the IL is confined in the narrow gaps between the individual polymer chains or clusters has proven to be a successful method of stabilizing ionic liquids in a polymeric materials. The polymer/IL composite-mix provides both physical and chemical interactions between the polymer and the IL, thus allowing the IL to be fixed in the polymer matrix made from a solvent casting procedure [51]. The synthesis of a composite membrane with an IL such as 1-Ethyl-3-methylimidazolium tetracyanoborate ([emim][B(CN)_4_]) for the effective capture of CO_2_ from a CO_2_/H_2_ and CO_2_/N_2_ mixture, was achieved [45]. The composite membrane consisted of a polymeric blend of poly(ethylene oxide) (PEO) and poly vinylidene fluoride (PVDF). The resulting permeability was as high as 1778 barrer with a corresponding selectivity of 41.1 for CO_2_/N_2_. Qiu et al. [46] synthesized a composite polymer material with an IL known as 1-butyl-3-methylimidiazo-lium bis[trifluoromethyl)sulfonyl]-imide [Bmim][Tf_2_N] for CO_2_ capture; the composite material comprised of poly(amide-6-b-ethylene oxide). At a temperature of 285 K and a pressure of 0.7 mPa, the estimated permeability and selectivity were 130 barrer and 17.5.

#### 1.3.3 Ionic liquid mixed matrix membranes (ILMMMs) composite

As stated previously, the limitation of CO_2_ capture in polymers owing to their permeability-selectivity differences, is one of the major drawbacks for membrane adoption in gas separation systems [52, 53]. Therefore, efforts are currently being made to solve this difficulty by developing polymeric materials that combine the adaptable features of polymers with gas separation capabilities when blended with inorganic materials [54]. Mixed matrix membranes have been explored by scientists as a suitable means of capturing CO_2_ [55]. Specific artificial fillers are added into some polymeric matrices to create mixed matrix membranes (MMMs). The MMM idea has the advantage of combining the simplicity of producing polymer films with excellent selectivity and permeability characteristics as evident in inorganic materials. This method has been tested with a variety of inorganic materials including zeolites, and metal-organic frameworks [54]. Challenges in the development of MMMs include filler dispersion, manufacture of defect-free membranes and cost-effectiveness. By combining MMMs with room temperature ionic liquids (RTILs), researchers have discovered a system that sufficiently improves CO_2_ capture efficiency and effectiveness whilst eliminating some issues associated with using MMM only. Huang et al. [56] synthesized a mixed matrix membrane in conjunction with graphene oxide and ionic liquid (1-(3-aminopropyl)-3-methylimidazolium bromide) for CO_2_ capture from a CO_2_/N_2_ mixture. The experiment was carried out at a temperature of 298 K and 4 bar. The resultant permeability was measured as 900 GPU. Bhattacharya and Mandal [53] synthesized a mixed matrix membrane by blending poly (ether-block-amide) (PEBA) and an ionic liquid for CO_2_ capture from a CO_2_/H_2_S mixture. The ionic liquid used was 1-ethyl-3-methylimmidazolium-ethylsulfate [EMIM][EtSO_4_]. The process conditions were 303 K, a pressure of 7 kg/cm^2^ and the resultant permeability of the synthesized material was recorded as 55 barrer.

#### 1.3.4 Poly(ionic liquid)s membranes (PILMs)

Another way to use integrated ILs for capturing CO_2_ is to make solid poly-ionic liquid (PIL) membranes from IL monomers using a direct polymerization technique [57, 58]. The Polymerization of IL and the construction of dense films of composite membranes comprising of poly-(IL) linkages provide huge benefits for the IL without any drawbacks anticipated with respect to the composite SILMs. To make gas selective membranes, the IL can be produced as a monomer and then polymerized [57]. SILMs are usually confronted with issues regarding IL-seepage from porous membranes at a certain differential pressure, thus imposing some process difficulties. However, as compared to commonly used polymeric membranes, they give larger mass fluxes of separated compounds which are accompanied with high separation factors [59].

**Figure 3:**
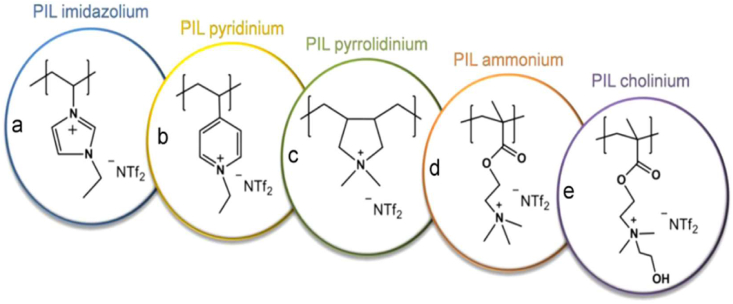
Schematic view of several poly(ionic)liquids. Adopted from Tomé et al. [60].

As presented in Figure 3, Tomé et al. [60] described the structures of five different PILMs namely, (a) poly([1-vinyl-3-ethyl-imidazolium][bis(trifluoromethylsulfonyl)imide]) (poly([ViEtIm][NTf_2_])), (b) poly([1-vinyl-3-ethyl-pyridinium][bis(trifluoromethylsulfonyl)imide]) poly([ViEtPy][NTf_2_]), (c) poly([1-vinyl-3-ethyl-pyrrolidinium][bis(trifluoromethylsulfonyl)imide]) poly([Pyr11][NTf_2_]), (d) poly([1-viny-3-lethyl-ammonium][bis(trifluoromethylsulfonyl)imide]) poly([EMTMA][NTf_2_]), and (e) poly([1-vinyl-3-ethyl-cholium][bis(trifluoromethylsulfonyl)imide]) poly([EMCh][NTf_2_]). The PILMs were seen to have better functional properties, endurance and structural strength; thanks to their polymer macrostructures.

Various solutions have been prepared to improve the transport properties of PILMs in order to overcome their low gas permeability and diffusivity [59]. Despite the several advantages of using PILMs for mixed gas systems at elevated pressures, little is known regarding the permeation behavior of these glassy poly-ILs. Thus, it is unclear as to how a strongly sorbing gas like CO_2_ influences its own movement (auto-plasticization) or that of a slowly diffusing constituent like methane [59, 61]. Considering some authors’ notion on the subject, the transport properties of poly-(IL) at increased CO_2_ feed pressure are also poorly understood [61]. Zarca et al. [62] synthesized a polymer composite (Poly([C_4_vim][Tf_2_N])-[C_4_mim][Cl]) with an IL for CO_2_ capture. The IL utilized was 1-vinyl-3-butylimidazolium bistriflimide. The experiment was carried out at 293 K, and the CO_2_ permeability was 5.2 barrer. Tomé et al. [60] synthesized a membrane composite together with an IL for CO_2_ capture. The ionic liquid used was 1-ethyl-3-methylimidazolium tricyanomethanide ([C_2_mim][C(CN)_3_]). The composite material comprised of Poly([Pyr11][C(CN)3]); the experiment revealed CO_2_ permeability of up to 542 barrer.

#### 1.3.5 Ionic liquid gel membranes (ILGMs)

Ionic liquid gel membranes (ILGMs) are a new technique for boosting the effectiveness of membrane materials [15, 59]. Integrating synthetic nanoparticles into polymer/IL matrices to make ILGMs has proven to be a cost-effective and a simple way of mitigating issues related to polymer chain packing, thus causing an increase in the free volume of the material [63]. Because gelled membranes may be made into thin sheets and packed into big modules for easy installation, utilizing polymers as gelling agents appears to be very appealing [64]. Considering the current state of arts on the subject, only a few studies on the gas transport properties of membrane-based ILGMs are available in literature with plausible dearth in ways of advancing these technologies for optimal gas purification and CO_2_ sequestration [15, 59, 65].

Moghadam et al. [65] synthesized an ionic liquid-membrane system for CO_2_ capture from a CO_2_/N_2_ mixture. The ionic liquid used was tetrabutylphosphonium prolinate ([P_4444_][Pro]). The gel membrane was amino acid ionic liquid-based. The experiment was carried out at a pressure of 0.1 kPa and a temperature of 303 K. The results showed that the permeability was about 52000 barrer with a selectivity of 8100. Mahdavi et al. [59] synthesized a composite polymer material together with an IL for CO_2_ capture from CO_2_/CH_4_ mixture. The ionic liquid used was 1-butyl-3-methylimidazolium hexafluorophosphate ([BMIM][PF_6_]). The composite membrane consisted of silica and polyether block amide (Pebax1074). The experiment was carried out at a temperature of 298 K and pressure range of 2–8 bar. The results showed that the permeability and selectivity are 153.6 barrer and 18.5 respectively.

**Figure 4:**
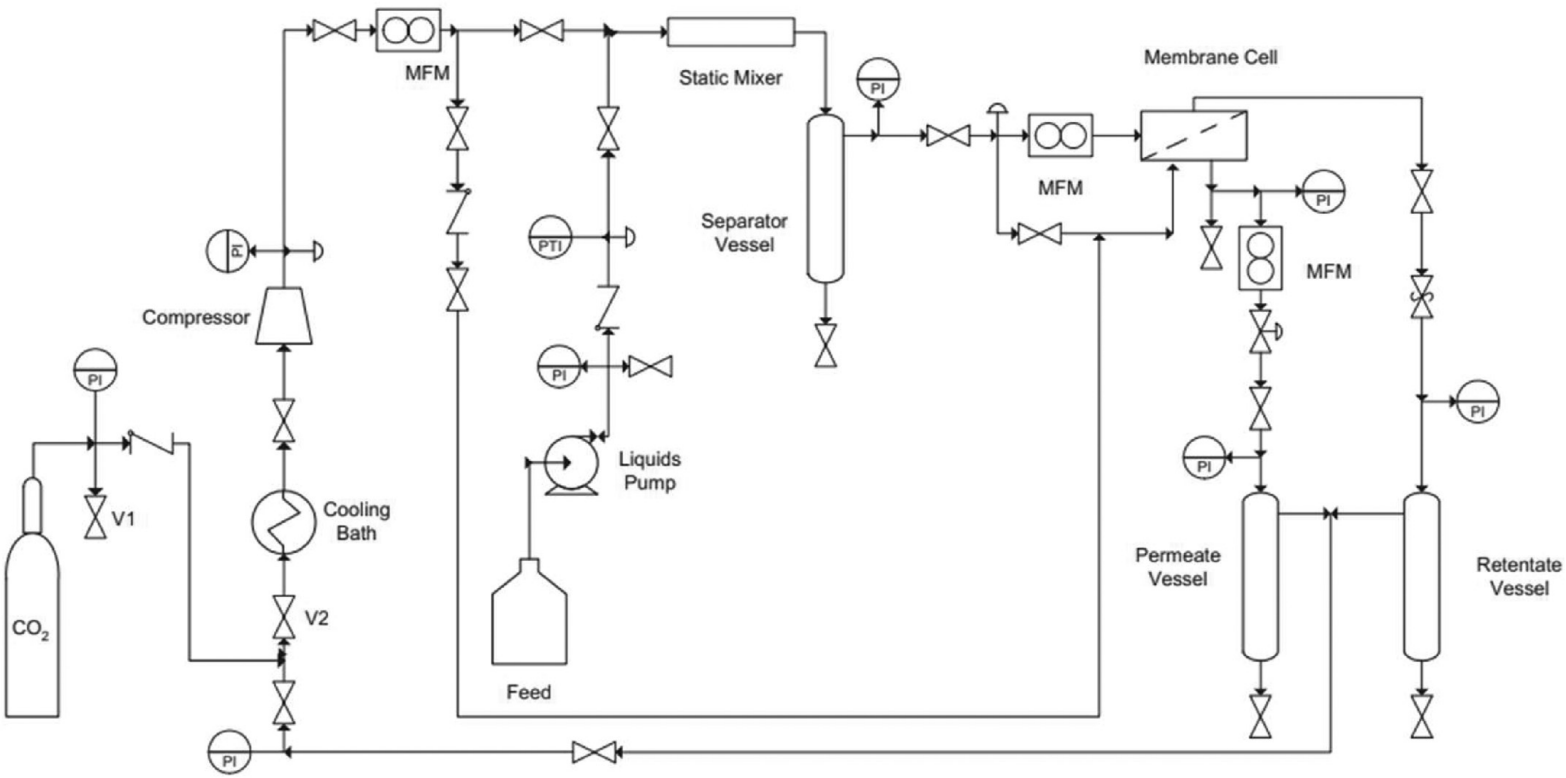
Carbon capture system consisting of an ILGM. Adopted from Couto et al. [63].

A carbon dioxide capture system was described by Couto et al. [63] as illustrated in Figure 4. The system consists of a permeate vessel, retentate vessel, feed tank, CO_2_ collector tank, a cooling bath, gas compressor separator vessel and the membrane (ILGM) vessel. Pressure, temperature and flow-rates were measured in the process. The gas-liquid feed was sent into the feed tank, after which it was allowed to flow into the separator-tank. With appropriate heating, the flue gas was received and sent to the membrane-tank where the gas was contacted with the membrane. The retentate gas (rich in CO_2_) was then stripped and sent to the retentate vessel. The permeate gas (lean in CO_2_) was transported to the permeate vessel. Thereafter, the permeate gas was sent to the feed tank for recycling purposes while the retentate gas was transported to the CO_2_ collector tank.

#### 1.3.6 Ionic liquid membrane contactors (ILMCs)

Membrane contactors are a composite technology that combine membranes and ionic liquids in special contact modes [66, 67]. It has several benefits including high interfacial area per unit volume, enhanced flow-rate, reduced equipment dimensions and cost-effective operations over a traditional packed column for CO_2_ absorption/stripping [68]. It also avoids issues like flooding and foaming that are common with traditional absorption equipment [69]. The absorbent for a CO_2_ capture membrane contactor must meet a number of criteria including thermal and chemical stability, low viscosity, low corrosion rate, high CO_2_ solubility, and selectivity over other gaseous species by tuning their selective gas permeation tendencies/properties [70]. A wide number of CO_2_ absorbents have been investigated in membrane contactors for flue gas purification in recent years [69]. In addition, ILs outperform standard membrane contactor-solvents, not only in terms of thermal stability at higher temperatures, but also in terms of the relative volatilities of the liquids, thus resulting in less solvent loss and lower energy use [71]. Maintaining membrane and sealing materials for a membrane contactor at higher temperatures is expensive and difficult [67]. Albo et al. [66] synthesized a membrane contactor and ionic liquid system for CO_2_ capture from a CO_2_/SO_2_ mixture. The ionic liquid used was 1-ethyl-3-methylimidazolium ethyl-sulfate. The experiment was conducted at a temperature of 288 K. The adsorption efficiency was recorded as 0.28. Similarly, Gomez-Coma et al. [72] used a membrane contactor and an ionic liquid for CO_2_ capture from a CO_2_/N_2_ mixture. The ionic liquid utilized was 1-ethyl-3-methylimidazolium acetate ([emim][Ac]). At a temperature of 348 K, the adsorption capacity was 0.45.

In lieu of the wide literature consulted, no study seems to have considered harnessing the reasons behind the poor performance of some membranes and ionic liquids for CO_2_ capture. However, since ionic liquid-membrane hybrid systems have been proven to hold great prospects for CO_2_ capture, it then became necessary to consider combining poorly performing membranes/ionic liquids and moderately suitable systems for carbon capture. This is because, it is believed that the induced functionalization of the membranes with the ionic liquids bring about property-modifications of the hybrid systems which in turn boosts their overall carbon capture potential.

**Figure 5:**
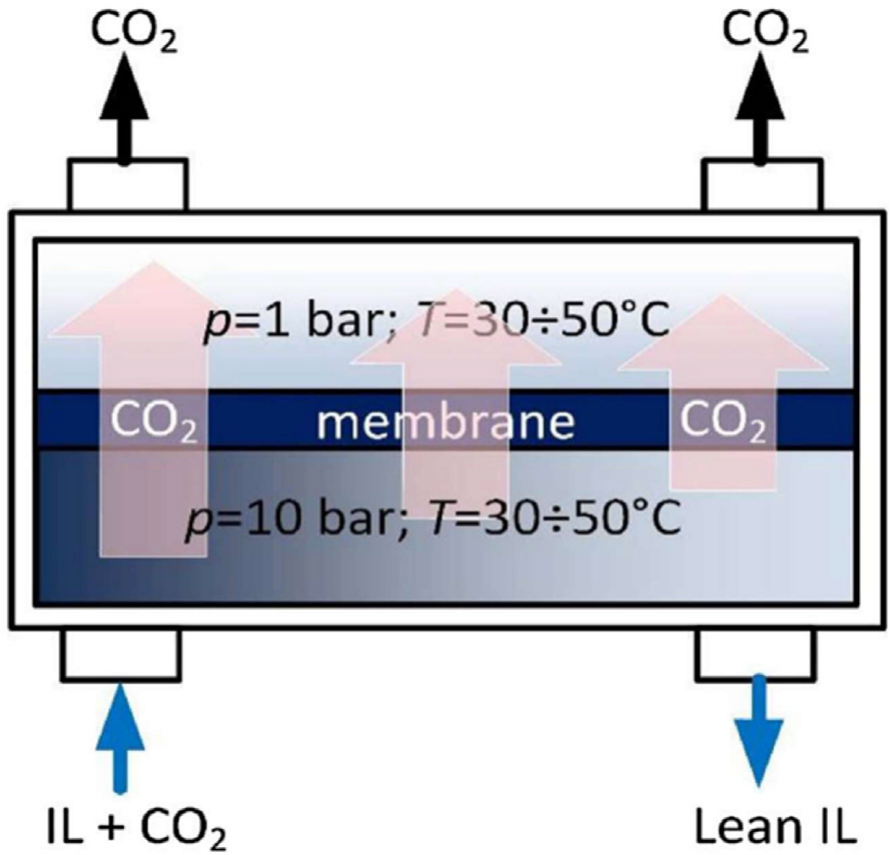
Composite membrane contactor and IL-CO_2_ capture system. Adopted from Bazhenov et al. [68].

Bazhenov et al. [68] described a CO_2_ stripping system that uses a composite membrane contactor as illustrated in Figure 5. The system consists of a support (stainless steel), thermal chamber for temperature regulation, pipe (steel) for heat exchanging and the composite membrane contactor. IL containing captured CO_2_ was passed through the bottom of the system so that it comes in contact with the contactor at a pressure of 10 bar and at low temperature. The CO_2_ was stripped and at a lower pressure of say 1 bar and higher temperature, the CO_2_ was released from the contactor and sent to a retentate collector. The lean IL consisting of reduced CO_2_ content was collected at the base of the retentate collector by means of a permeate collector.

## 2 Challenges associated with choosing membrane-ionic liquid systems for gas separation

There are several challenges that scientists may encounter when setting up a novel system comprising of hybrid membrane-ionic liquid systems. They include:

• Cost of starting material: The costs of the materials for most membrane-ionic liquid systems are quite high. Due to the rarity of some of the starting materials, and also the demand for these materials, the cost of these materials may be exorbitant. The cost of importing some of these materials is also high, hence the need to search for viable alternative ionic liquids of moderate costs as well as the need to optimize their required quantities for use.
• Availability of the starting materials/precursors: Some of the materials required for the experimental procedures may be scarce, hence, access to these materials may require some forms of importation which involves excessive costs, thus, it is necessary to begin to look into sourcing some of these materials locally.
• Methods of synthesizing the desired ionic liquids and membranes: For membrane synthesis, the most popular method is the phase inversion method which involves the controlled polymer change from liquid phase to solid phase. Ionic liquid preparation also requires mixing of different chemical solutions using a given set of guidelines. The method of synthesis is important when considering the type of membrane-ionic liquid system to be used.
• The method of blending the chosen membranes and ionic liquids prior membrane casting: The most used method for the integration of ionic liquids in membranes is the solution casting method, where the liquid is premixed with the membrane solution prior casting. The membrane pellets are mixed with a solvent solution, such that it undergoes sonification, wherein it is doped with the ionic liquid and another dopant (i.e., ethanol), and poured unto a watch-glass upon which a glass rod or mechanical process is used to configure its shape into a solid membrane-ionic liquid composite. The method is important in determining the quality of the hybrid adsorbent to be produced.
• Measure of compatibility between membranes and ionic liquids: The nature of the ionic liquid and the membrane selected for hybridization are important in determining the compatibility of both materials. The transport properties, activity coefficients and hydrodynamics of both materials are also major determinants of how compatible they are with each other.
• Selectivity of the different components in the blends: in determining the membrane and ionic liquid to be combined, selectivity is an important factor. Selectivity involves the permeation efficiency of one component/gas against another (or others) within a gaseous mixture. For CO_2_ capture, it is important that the product material has good selectivity for CO_2_ compared to other component gasses in the bio-/flue gas. Ionic liquids with low selectivity are expected to be paired with membranes of high selectivity to enhance their performance and vice-versa.
• Composition or ratio of components in the blends: for the preparation of the desired hybrid material of membrane-ionic liquid systems, there must be a balance in the concentration of the component materials for the optimum performance of the formed product materials.
• Operating conditions: These are usually the temperature and pressure conditions adopted while preparing the desired product materials. Preparing materials at temperatures above stipulated guidelines in literature may yield negative effects, thus leaving the material damaged.
• Membrane permeability and porosity: morphology, surface area and pore structures determine the permeability of CO_2_ in membranes. If the membrane does not meet the structural permeability requirements, it will be ineffective in capturing CO_2_ from bio/flue gases. However, this property has to be carefully moderated with the selectivity of the hybrid material.
• Chemical, hydro and thermal stability of the hybrid-system: the properties of the adsorbent material that allows it to be used in different environments include its resistance to fouling caused by water, harsh chemicals and the threshold properties of the materials at high temperatures, all of which are important in determining its gas separation efficiency/performance and quality.
• Polarity of the ionic liquid: The pairing of anion and cations in ILs offer some measures of hydrophobicity and polarity, which are highly essential for CO_2_ capture. The cation plays a minor role in determining the CO_2_ capture performance of the ionic liquid, whereas, the strategic placement/distribution of the anion in the hybrid material can offer varying levels of absorption capacities that can be beneficial to the overall system.
•Nature of the ionic liquid and membrane: The type of ionic liquid and nature of the membrane are important in the preparation of a hybrid material. Poor performing membranes may not be compatible with some high performing ionic liquids, and vice-versa. Although the physical properties and chemical compositions of membranes and ionic liquids may influence their use, it then becomes necessary to have a good understanding of the inherent characteristics of the individual components for optimal performance of the synthesized composite-adsorbents.

## 3 Flow scheme of the proposed ionic liquid-membrane system for carbon dioxide capture from a post-combustion process using biomass as feed

### 3.1 General description

The schematic presentation of the proposed ionic-liquid membrane carbon capture and storage system is as illustrated in Figure 5. The columns and storage vessels are made of high carbon steel. Biomass (Napier grass) is supplied to the feed tray of the combustor. Heating is provided by connecting an electric power source to the heater at the bottom of the combustor until the temperature rises to 500 °C. The gas generated from the combustor eludes at the top of the column and passes through series of air fin coolers with air in circulation at high flow rate to bring the temperature to about 30–45 °C. A filter is attached to the exit line from the cooler which helps to remove particulate matter from the biogas before it enters the adsorber containing the adsorbent (ionic liquid-membrane system) where the gas makes contact with the adsorbent. A sample point is available at the inlet section of the adsorber which is to be used to take a sample of the gas for analysis in order to ascertain the composition of CO_2_ in the biogas. After stripping the gas of CO_2_, the resulting effluent/permeate gas is received at the top of the adsorber where the CO_2_ concentration is again measured by taking gas sample from a sample point in order to ensure that the CO_2_ concentration in the gas is brought to a permissible limit. However, upon stripping the biogas of CO_2_, if the CO_2_ concentration is still above the required limit, the gas is recycled back to the column for further stripping. Once, the permeate gas is almost/totally free from CO_2_, the permeate gas is sent to the storage-facility. Thereafter, the adsorbent is recovered for regeneration; this entails a desorption process that is inclined to solvent/temperature swing desorption. The regenerator releases the ionic liquid-membrane-desorbed CO_2_ via a pressure relief valve which responds to CO_2_ partial pressure within the limit of 2 bar. The efflux-CO_2_ is then compressed via a compressor before it is sent to the CO_2_ storage tank.

Mass flow controllers, pressure gauges, and flow meters are installed on the columns to monitor and measure stream flow rates and pressures. The adsorption column is packed with a sample-composite membrane-ionic liquid adsorbent. Considering the benefits of PILs for CO_2_ capture, the membrane-ionic liquid system makes use of PIL as the desired IL. The collection tank is mounted above the desorption column. The storage tanks are connected to both the collection/recycling and desorption tanks. A gas filter is used to filter off particles in the bio/flue gas.

### 3.2 Detailed procedure

**Figure 6:**
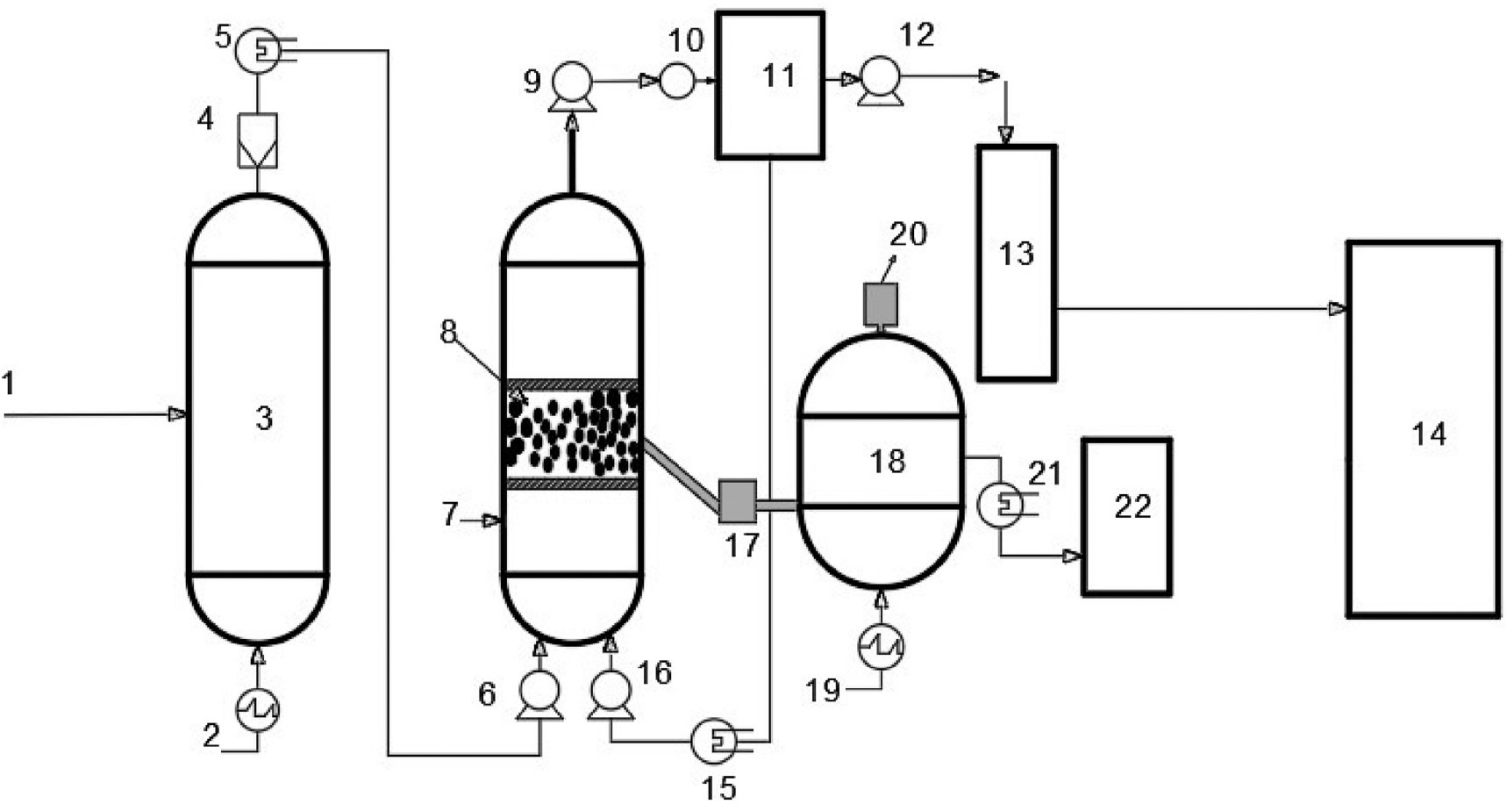
Schematic section of the proposed ionic liquid-membrane carbon dioxide capture system.

Note: Other byproducts of the combustion process include biochar and bio-oil which may be drawn from the base of the combustor.

Biomass is fed (1) into the combustor and heated using a power source (2) to release bio/flue gas containing CO_2_. Desulphurization and denitrification units are used to pre-treat the gas entering the adsorption column. From the combustor (3), the exhaust gas is sent into the gas filter (4) where it is filtered, initially compressed using a compressor (5), isobarically cooled, using an air coolant (6) in the range of 35–40 °C and sent into the adsorption column (7). Water vapor from the gas is condensed before entering the adsorber, thus allowing for overall water balance. The fast gas adsorption kinetics is favored by the inherent low temperature in the adsorber. The flue gas/biogas comes in contact with the hybrid adsorbent (8). The permeate gas (lean in CO_2_ concentration) leaving the hybrid adsorbent exits through the top of the adsorption column, is compressed using a compressor (9), passes through a valve (10) and flows into the collection tank (11). The gas is further compressed by a compressor (12), sent into the second collection (13) tank and finally sent to the storage tank (14). If the permeate gas still contains a significant amount of CO_2_ (in the first collection tank), it is cooled by an air fin cooler (15), compressed by a compressor (16) and sent to the adsorption tank for re-contacting with the hybrid adsorbent. A conveyor (17) sends the used hybrid adsorbent to the desorption column (18). At the desorption column, a heating source (19) is used to heat the used hybrid adsorbent to temperatures as high as 250 °C so as to ensure that the retentate gas (rich in CO_2_ concentration) is removed. A pressure valve (20) helps to indicate the presence of CO_2_ gas. The CO_2_ gas is cooled with air (21) and sent to the storage tank (22). Figure 6 is an illustration of the proposed postcombustion carbon capture system/scheme comprising of an integrated membrane-ionic liquid-nanoparticulate composite for effective CO_2_ capture:

## 4 Future prospects of membrane-ionic liquid technologies for carbon capture

Several materials for CO_2_ capture are being explored on a large scale [73]. Membrane technology is a tried-and-true method of sweetening natural gas. For example, in 2009, it had a 20 % share of the CO_2_/CH_4_ separation market. Novel gas separation opportunities in the petrochemical and refining industries, as well as other post-combustion CO_2_ capture applications, not only provide new opportunities for membrane separation, but also necessitate the development of high-performing membrane materials [74]. However, the unexplored separation prospects in petrochemical refineries, as well as other post-combustion CO_2_ capture applications, need the development of high-performing membrane materials. The recorded carbon dioxide adsorption capacity was 90 % of the initial cycle capacity after ten adsorption/desorption cycles [75]. For the rate-controlling step, the hybrid adsorbent had an adsorption capacity of 143 × 10^−3^ mmol g^−1^. Many types of porous adsorbents, such as metal–organic frameworks (MOFs), covalent organic frameworks (COFs), zeolites, and carbon-based nanomaterials have been produced and studied for CO_2_ gas collection to date [76, 77]. However, MOFs have long been thought to be the finest candidates for CO_2_ storage. At 298 K and 14 bar, the quantity of CO_2_ absorbed by MOF-5 was reported to be as high as 48.0 wt.%. Functionalizing ligands and introducing them into open metal sites can further boost the CO_2_ storage capacity of the material [78]. Permeability is the amount of gas molecules that can flow through a membrane in a unit stream. It is normalized by parameters such as pressure, temperature and thickness. Molecule-permeation through membranes is caused by a difference in chemical/activity/fugacity potential [79]. Furthermore, conventional CO_2_ capture systems that employ aqueous amine solutions have several drawbacks ranging from insufficient CO_2_ sorption, corrosiveness, poor thermal stability, large solvent losses, and high energy consumption during regeneration. As a result, the creation of a highly efficient technology/adsorbent is critical [80].

**Figure 7:**
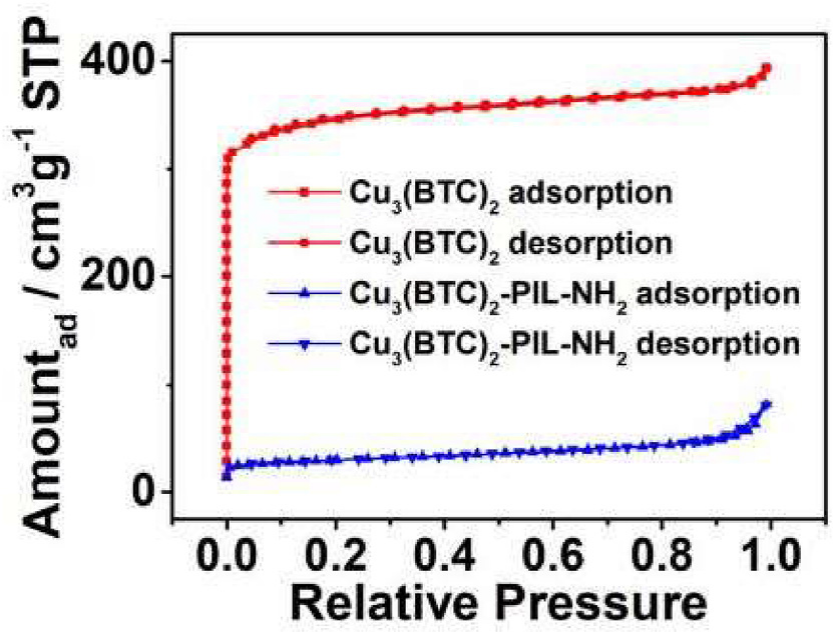
BET analysis of multiple PILM systems. Adopted from Yang et al. [83].

**Figure 8:**
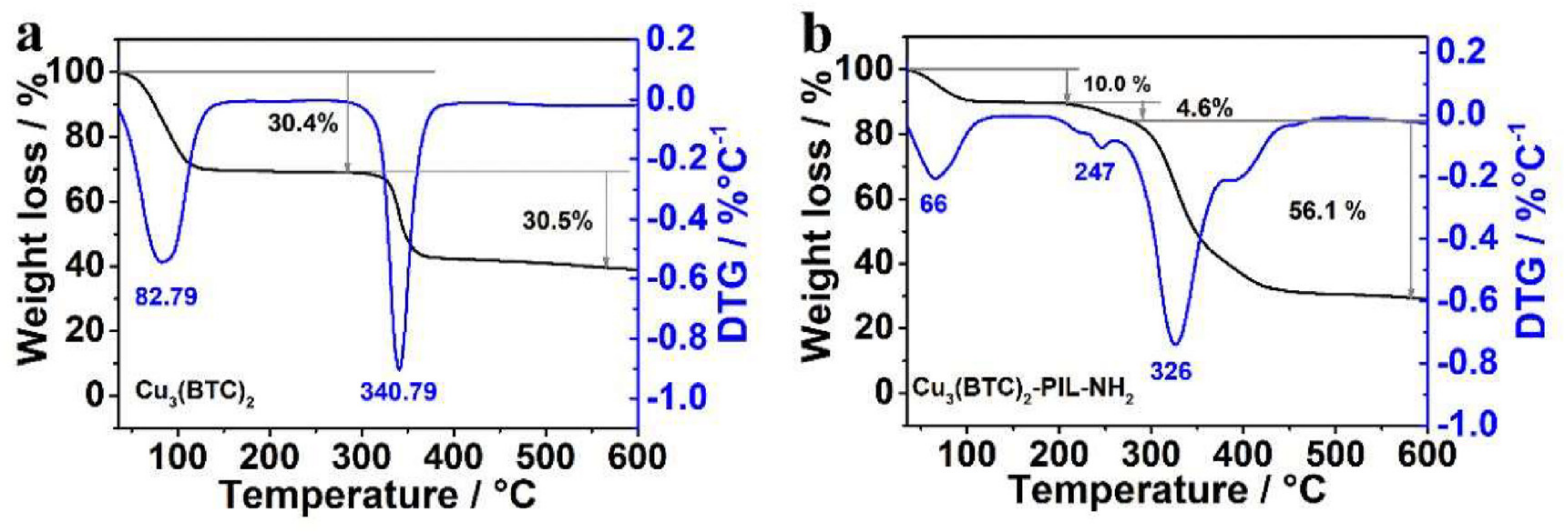
TPD analysis of multiple PILM systems. Adopted from Yang et al. [83].

At temperatures below 100 °C, ILs are characterized as liquid organic salts. A wide liquid range, minimal vapor pressure, excellent thermal stability, flexible features, and above all, high CO_2_ affinity are some of the unique qualities of ILs [80]. On the other hand, the high viscosity of ILs, is a significant disadvantage. This flaw, however, can be remedied by using the right cations and anions. Thakur et al. [81] found that [bmim][BF_4_] has a viscosity of 79.5 cP, which is higher than that of monoethanolamine. Because of the inherent high viscosity of the liquid, molecular diffusion is slow while the equilibrium time lengthens, thus limiting the rate of absorption. Under atmospheric pressure, Mirzaei et al. [82] examined the CO_2_ sorption capabilities of silica-supported 1-butyl-3-methylimidazolium-based ionic liquids (ILs). It was discovered that [Bmim][BF_4_] [Bmim][PF_6_], and [Bmim][Tf_2_N] as a result of their high viscosities recorded decreased CO_2_ capture at an increase in temperature from 25 °C to 50 °C. 0.52 wt% [Bmim][TfN] [Bmim][PF_6_] of 0.41 wt % and [Bmim][BF_4_] of 1.04 %, all had relatively good CO_2_ sorption capacities with a lot of room for improvement. To improve the performance and efficiency of ILs, they can be joined covalently to form polymeric ionic liquids (PILMs) [80]. Polymers of ILs that are covalently bound are known as PILs. PILs, like ILs, have a minimum of one ionic center. PILs frequently contain cations such as imidazolium. When an IL is added to a PIL, its CO_2_ permeability and CO_2_/N_2_ Perm-selectivity can be increased to levels greater than the Robeson limit specifications (selectivity and permeability) [73]. A sample-BET curve (Figure 7) and TPD curve (Figure 8) are presented for the Cu_3_(BTC)_2_ and Cu_3_(BTC)_2_-PIL-NH_2_ respectively. The characteristic features obtained from BET analyses are also included in Table 2.

**Table 2:** BET characterization of Cu_3_(BTC)_2_ and Cu_3_(BTC)_2_-PIL-NH_2_.

Samples	Samples Surface Area (m^2^∙g^−1^)	Samples Pore Volume (cm^3^∙g^−1^)	Samples Pore Diameter (nm)
Cu_3_(BTC)_2_	1352	0.60	1.8
Cu_3_(BTC)_2_-PIL-NH_2_	107	0.12	4.5

In the BET curve (Figure 7) shown for Cu_3_(BTC)_2_-PIL-NH_2_ and Cu_3_(BTC)_2_, porous structures of both sorbents were seen to have significant influence on their gas adsorption behaviors. The characteristic nitrogen adsorption-desorption isotherms observed for the porous Cu_3_(BTC)_2-_PIL-NH_2_ and Cu_3_(BTC)_2_, show that Cu_3_(BTC)_2_ exhibited Type I isotherms, thus confirming the presence of micropores. With the grafting of PILs onto the adsorbent's surface, the geometry of the isotherm appeared significantly modified at low relative pressure, thus suggesting a significant drop in the number of micropores caused by the blockage of the surface-attached polymer chains [83]. Table 2 shows the characteristic porous features such as surface areas, pore volumes, and average pore sizes of the adsorbents. The Cu_3_(BTC)_2_ has a surface area of 1352 cm^2^ g^−1^ with corresponding average pore diameter of 1.8 nm and pore volume of 0.61 cm^3^ g^−1^. However, the surface area and pore volume decreased drastically to 107 m^2^ g^−1^ and 0.12 cm^3^ g^−1^, respectively owing to the intensive coverage of micropores on Cu_3_(BTC)_2_ induced by the polymer chains. In addition, the increased average pore size of the adsorbent after complexation with PIL-NH_2_ is likened to the presence of pore-slits or pore formation resulting from the accumulation of the PIL-NH_2_-modified particles.

In Figure 8a and b, CO_2_ adsorption characterization was carried out using Temperature programmed desorption (TPD technique) at 25–200 °C of pre-adsorbed molecules of CO_2_ in the Cu_3_(BTC)_2_ and Cu_3_(BTC)_2_-PIL-NH_2_ respectively at 25 °C and CO_2_ pressure of 0.2 bar for 2 h. The Cu_3_(BTC)_2_ (Figure 8a) and Cu_3_(BTC)_2_-PIL-NH_2_ (Figure 8b) TPD response curves show only one desorption peak at about 95 °C for Cu_3_(BTC)_2_ with corresponding CO_2_ adsorption capacity of about 3.09 cm^3^∙g−^1^. The observed desorption peak can be allotted to the desorption of physically sorbed CO_2_ molecules and adsorbed CO_2_ concentrations via weak interactions with copper ions in Cu_3_(BTC)_2_ [83]. For Cu_3_(BTC)_2_-PIL-NH_2_, there are two desorption peaks at about 96 and 200 °C, respectively. Furthermore, comparing Cu_3_(BTC)_2_ and Cu_3_(BTC)_2_-PIL-NH_2_, it can be said that an additional weight loss region between 210 and 275 °C was observed, which is attributed to the evolution of water molecules that are tangled with N atoms in the PIL via hydrogen bonding. In essence, one can affirm that the synthesized Cu_3_(BTC)_2_-PIL-NH_2_ has a good thermal stability below 200 °C.

Another group of ILs that have drawn significant interests are RTLs, because they may be tailored to have high CO_2_ affinity. At room temperature, RTILs are substances that remain liquids. Also, the class of liquids tagged protic ionic liquids (PrILs) have several benefits over ordinary ILs, including low corrosivity, excellent thermal stability, insignificant vapor pressure, low thermal expansion, low cost, ease of synthesis, high CO_2_ absorption capacity and selectivity [73]. Fang et al. [84] examined four distinct PrILs comprising of 1-vinyl-3-butylimidazolium ([VBIM]^+^) and four different anions, including hexafluorophosphate ([PF_6_]) and bis(trifluoromethylsulfonyl)imide ([TF_2_N]). At 308 K and infinite dilution, the sorption and diffusion of CO_2_ and N_2_ in the four membranes were investigated. Based on the investigation, both Poly([VBIM][TF_2_N]) and Poly([VBIM][PF_6_]) had good diffusivities and solubility coefficients. In addition, atomic simulations of the IRMOF-1 supported ionic liquid (IL) membrane CO_2_ capture were performed by Gupta et al. [85]. The ILs had the same cation, 1-n-butyl-3-methylimidazolium [BMIM]^+^, but four separate anions: hexafluorophosphate [PF_6_], tetrafluoroborate [BF_4_], bis(trifluoromethylsulfonyl)imide [Tf_2_N], and thiocyanate [SCN] in their combinations. At a pressure of 100 kPa, the solubility coefficient, permeability and diffusivity of the [BMIM][SCN]/IRMOF-1 membrane were found to be 0.642 [cm^3^ (STP) cm^−3^ (membrane) (cm Hg)^−1^], 40 118 barrer and 6.25 10^−6^ cm^2^ s^−1^, respectively.

Recently, few studies were published on the application of ILs in gas separation processes. Substantially enhanced CO_2_ permeability and selectivity can be attained simultaneously when polymer matrices and IL additives are used. The IL is thought to function in these hybrid membranes as a plasticizer that causes the host polymer to swell, thus increasing CO_2_ diffusivity and, as a result increases CO_2_ permeability. The chemical affinity between CO_2_ and IL, especially if the IL contains CO_2_-philic functional groups, is what causes the accompanying high CO_2_ selectivity [86]. Nexar et al's [87] molecular architecture enables the creation of several property-governing nanoscale morphologies by allowing for chemical composition variation. They synthesized a composite membrane-ionic liquid for the separation of CO_2_ from natural gas. The experiment was conducted at a temperature of 308 K and a pressure of 100 kPa. The results showed a maximum selectivity and permeability of 128 and 194 barrer. The mass and thermal stability of the hybrid ILM was recorded at temperatures up to 723 K. Despite the positive results, the hybrid membrane-ionic liquid showed a trend of mass losses at temperatures slightly below the temperature of decomposition (Td). It also revealed an increase in water sorption ability with an increase in the amount of IL added. This can increase the hybrid ILM's susceptible to fouling. Furthermore, improved materials like MXenes have been used in experiments to create 2D layered ionic liquid membrane systems. These systems have a number of benefits, including ultra-fast water permeability, precise ion screening, and most significantly, remarkable gas separation performance [87]. They created a composite membrane-ionic liquid system for carbon capture from natural gas. The operating temperature and pressure were 298.15 K and 400 kPa respectively. The results showed high adsorption capacity and high regeneration capacity (4 cycles) with a regeneration efficiency of up to 98.7 %. A decrease in adsorptive capacity with an increase in temperature was however observed. Fang et al. [84] created a hybrid membrane-ionic liquid system for the capture of carbon from a CO_2_/N_2_ mixture. At a temperature of 323.15 K, the selectivity was given as 25. When functionalized with the fluorine (F) as functional group, the membrane was seen to exhibit high gas selectivity. Whereas, when it was functionalized with the hydroxyl functional group (-OH), the membrane displayed low gas selectivity. However, when the membrane was functionalized with oxygen (O) as functional group, the membrane exhibited average gas selectivity. Selectivity of absorbed molecules in the IL membranes with fluorine functional group was higher than those functionalized with both the –OH and –O functional groups. The structure and characteristics of these restricted ILs can be greatly altered, thus enhancing the stability and toughness of the entire system. Ionic liquid molecules can be immobilized on solid surfaces [87]. The combination of ILs and membranes such as zeolites, MOFs, MXenes, etc., can overcome many drawbacks associated with traditional adsorption-absorption techniques by expanding the contact area between the target-gas and ILs. However, fluids and solids typically behave as a continuum in existing macroscopic investigations that bother on separation systems, thus, the specifics of molecular interactions which are attributed to heterogeneity, have been overlooked. The liquid-solid interface and transport characteristics displayed by microfluidics are also greatly impacted by non-bonding interactions. The intricacy of MXenes' microstructure and laborious experimental procedures have a negative impact on the abilities of experimental methods to examine materials like MXenes in a systematic and efficient manner. Information at the molecular level that cannot be directly obtained experimentally can be directly provided through molecular simulations [87].

**Figure 9:**
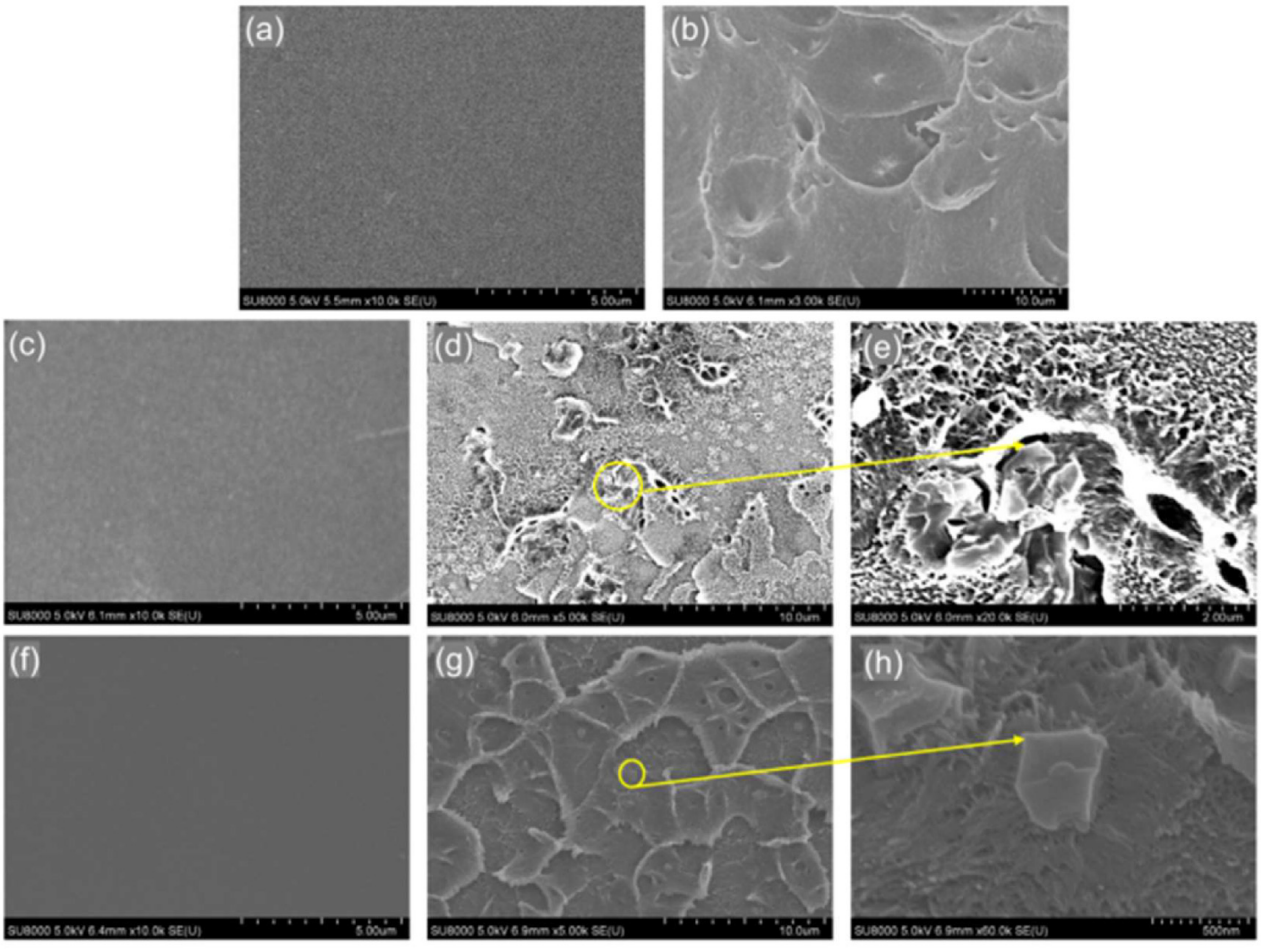
Surface morphology and cross-section of: (a, b) pure PIM-1 membrane @ 5 and 10 μm magnification respectively (c, d and e) 5 wt.% ZIF-67/PIM-1 MMM @ 5, 10 and 20 μm magnification respectively (f, g and h) 5 wt.% [HDBU][Im]@ZIF-67/PIM-1 MMM @ 5 μm, 10 μm and 500 nm respectively. Adopted from Han et al. [90].

Ionic liquid-membrane systems (ILMs) have recently been suggested as viable CO_2_ absorbent-adsorbent systems because of their excellent thermal stability, low volatility, and physicochemical stability. Both 2D nanochannel-confined ILs and ILs supported by porous matrix exhibit distinctive thermodynamic and kinetic features, which contribute to their effective CO_2_ separation and capture. Therefore, creating a technique for coating porous membrane materials bearing a surface likened to that of graphene with a thin ionic liquid layer which helps to enhance the dynamic control of pore size, is of great research interest [88, 89]. In addition, when the pore size of porous graphene reaches 7 Å, the [BF4] anions may not effectively act as gating agents owing to the fact that the pore size of 7 Å is too large for [BF4]^-^. Further increase in the thickness of the membrane may improve its selectivity with a resultant decrease in the permeability. Figure 9 shows the surface morphology and cross-sectional views of a doped PIM membrane with different weight-percent ionic liquids.

Due to their crystalline composition and microporous lattice structures, which demonstrate exceptional heat and chemical resilience, zeolites are remarkably desirable materials for heterogeneous catalytic processes [89]. In ref. [89] the synthesis of a complex membrane-ionic liquid material for CO_2_ capture from a CO_2_/CH_4_ mixture was carried out. The experiment was conducted at a temperature of 303 K and a pressure of 200 kPa. The results showed very good carbon capture efficiency. The results show that the HZSM-5 (H-Zeolite Socony Mobil-5) catalyst retained its structure throughout the absorption/desorption cycles. ZSM-5 (Zeolite Socony Mobil-5) exhibited the best performance among the examined specimens, and reduced the heat duty by 45 %–50 % compared with that of the catalyst-free system. The use of zeolites improved the desorption rate by 12–14 times compared to that of the blank non-aqueous MEA and IL-MEA solvent blend. However, a loss in catalytic activity was observed after the fifth desorption cycle, and the average catalytic stability was observed to be approximately 82 %. In another study, it was noted that, owing to the variation in fluid nature, high CO_2_ absorption capacity, and low viscosity of superbase ionic liquids (ILs) (a family of functional ILs), these ILs have the potential to act as interfacial wetting agents which in turn improve the compatibility and CO_2_ separation performance when used with MMMs [89]. The authors synthesized a hybrid composite membrane-ionic liquid system for CO_2_ capture from a CO_2_/CH_4_ mixture. The experiment was carried out at 303 K and 0.1 mPa. The maximum permeability and selectivity were shown to be 76 barrer and 104.3, respectively; a significant trade-off between selectivity and permeability of the CO_2_ molecule was noted.

**Figure 10:**
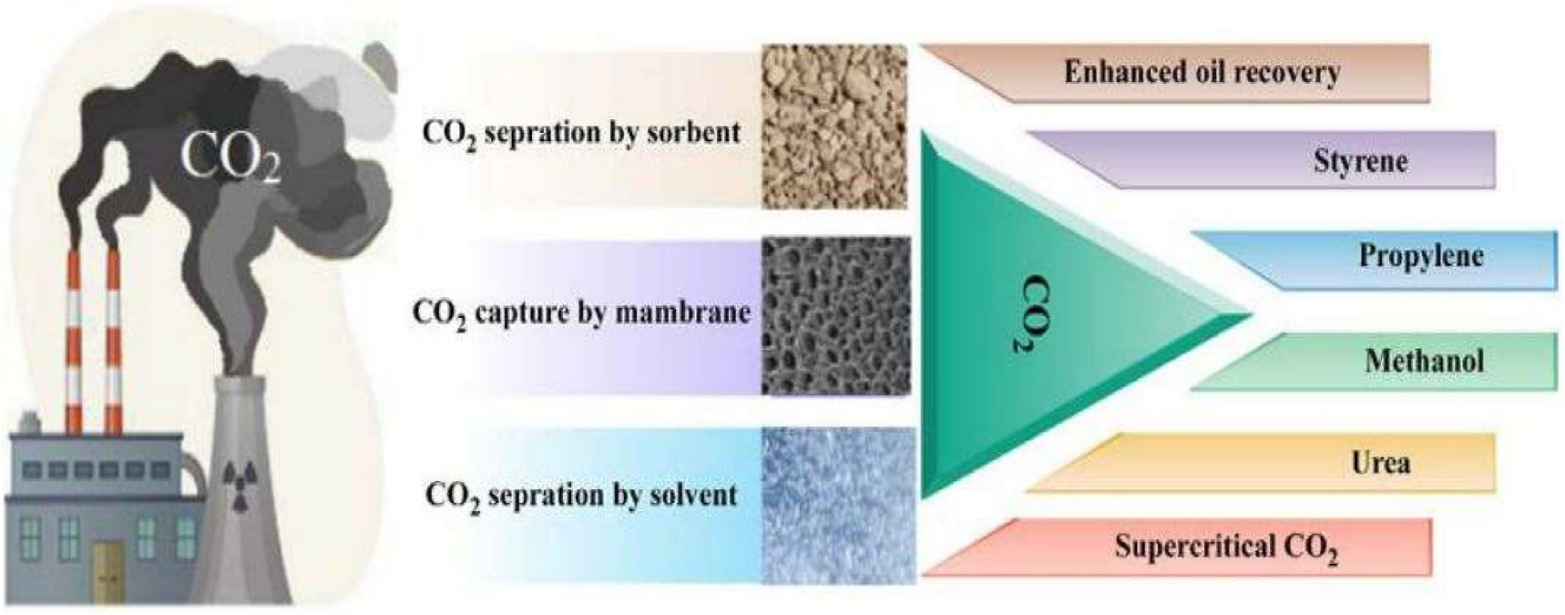
A simplified pathway for embracing a circular economy: CO_2_ production, capture/separation, storage. recycle/reutilization. Adopted from Meshksar et al. [133].

Again, considering Figure 9 a-h which is an illustration of the surface morphology and cross-sectional view of the pure PIM-1 membrane @ 5 (Figure 9a) and 10 μm (Figure 9b) magnification respectively, 5 wt.% ZIF-67/PIM-1 MMM @ 5 (Figure 9c), 10 (Figure 9d) and 20 μm (Figure 9e) magnification respectively, 5 wt.% [HDBU][Im]@ZIF-67/PIM-1 MMM @ 5 μm (Figure 9f), 10 μm (Figure 9g) and 500 nm (Figure 9h) respectively, it can be seen that IL can interact physically or chemically with GO when sandwiched between two pieces of graphene oxide sheets. As a result, all systems' cations-anions, GO-cations, and GO-anions interaction energies are usually calculated and experimentally determined. The GO-cations' interaction energy in the IL/GO system is higher than that of the GO-anions. Additionally, the energy of interaction between GO cations and anions is higher than that of GO and IL separately. The interaction energy of GO-cations/anions reduces as the layer of separation widens, thus the interaction energy of anions and cations increases [90, 91]. Additionally, it was discovered that the bulk ILs' anion-cation interaction energy is higher than that of the confined system. The restricted [Bmim]^+^ tends to interact significantly with the GO surfaces via electrostatic interactions, because GO surfaces are negatively charged, which in turn lowers the anion-cation interaction energy. The lower CO_2_/CH_4_ diffusion rate in IL/GO systems compared to those of the bulk IL, is likely caused by the higher interaction between the CO_2_/CH_4_-confined IL and lower IL diffusion in the confined GO. Additionally, as the GO layer spacing rises, the diffusion rate of the IL increases as well which facilitates the diffusion of CO_2_/CH_4_ and increases the rate at which it occurs. Additionally, in all the simulated systems, CO_2_ diffuses at a somewhat faster rate than CH_4_. In summary, the IL channels in the IL/GO system contribute more to the diffusion of CO_2_, which is a reflection of the membrane's selectivity for CO_2_ [91]. The outcome supports the fact that confinement decreases gas self-diffusivity through/onto a porous structure but does not decrease its selectivity. As a result, it was discovered that the interaction energies of CO_2_ with anions are significantly higher than those of CH_4_, while the difference in diffusion rate between CO_2_ and CH_4_ is not significant, with an indication that gas solubility plays a primary role while diffusion plays a secondary role. The effect of ILs/GO membrane-anions on CO_2_/CH_4_ separation was also examined since it is a well-known fact that anions play a major role in CO_2_ absorption for bulk ILs. A synthetic composite ionic liquid-membrane system for the capture of carbon dioxide from a mixture of CO_2_ and CH_4_ was referenced. The experiment was carried out at a temperature of 298 K and a pressure of 100 kPa. The results showed a maximum permeability of 4721 barrer and selectivity of 14.3. An increase in temperature led to a decrease in permeability. In addition, with an increase in the amount of [HDBU][Im]@ZIF-67, the temperature of decomposition (Td) decreased. Owing to the prospects inherent in the use of ionic liquids and other solid adsorbents, their consideration for integration in membranes is so as to improve their gas retention abilities beyond the capacities of the synergistic effects offered by the individual components in IL-solid systems lacking membrane-supports. Since, there are relatively scarce volume of literature that bother on IL-na-noparticulate-membrane systems, this review paper looks into the prospects of some membranes that have barely been tested with ionic liquids but have great potentials for CO_2_-sequestration. Table 3 contains information on some membrane-ionic liquid systems that have been adopted for CO_2_ capture while Table 4 provides information on the selectivities and permeabilities of some membranes.

**Table 3:** Conditions for application of membranes, ionic liquids, their strengths and weaknesses.

Membranes							
Type of membrane	AdsorptiveCapacity or carbon capture efficiency (%/ $\frac{{CM}^{3}}{g}$) (selectivity (-))	Conditions of application	Operating pressure	Strengths	Weaknesses	Selected Blends	References
		T (K)	P (kPa)				
m-DAC	29.8^b^	-	110	-	-	1. [P_66614_][4-Cl-PhO] 2. [P_66614_][4-NO_2_-PhO] 3. [E_3_Py] [NTf_2_] 4. [E_3_Py] [NTf_2_] 5. [aemmim][Tau] 6. [N_11_][Gly]+MDEA 7. [Emim][MDEGSO_4_]	[92]
PVAm/PSf HF	230^b^	298	300	1. Relatively average permeance 2. High selectivity	Low thermal stability	1. [C_4_Py][NTf_2_] 2. [E_3_Py] [NTf_2_]	[93]
PVAm/PSf HM	133^b^	298	160	1. Relatively average permeance 2. Relatively average selectivity	Low thermal stability	1. [C4Py][NTf2] 2. [E3Py] [NTf2]	[93]
PVAm/PPO	68^b^	298	120	1. High permeance 2. High selectivity	Low thermal stability	1. [C_4_Py][NTf_2_] 2. [E_3_Py] [NTf_2_]	[93]
Pebax /Zeolite Y	30^b^	330	10.324	1. High CO_2_ permeance 2. Good CO_2_/N_2_ selectivity	-	1. [E_3_Py] [NTf_2_] 2. [aemmim][Tau] 3. [N_11_][Gly]+MDEA 4. [Emim][MDEGSO_4_]	[94]
polyvinylamine (PVAm)/piperazine glycinate (PG)	143^b^	330	-	1. High CO_2_ permeance 2. High CO_2_/N_2_ selectivity 3. Excellent stability in the presence of SO_2_ and O_2._ 4. High thermal stability	High operational cost due to the higher amount of water required at higher temperatures	1. [C_2_OHmim][Gly] 2. [BMIM][PF_6_]-MEA 3. [EMIM] [BF_4_]-MEA 4. [P_66614_][4-NO_2_-PhO]	[95]
hydrophilic polymeric membrane based on microfibrillated cellulose (MFC)	500^b^	308	-	1. Increased aspect ratio 2. Excellent mechanical properties 3. Has a very good potential to form hydrogen bond 4. Good chemical stability and the ability to be used in humid environments	-	1. [C_2_OHmim][Gly] 2. [BMIM][PF_6_]-MEA 3. [EMIM] [BF_4_]-MEA 4. [P_66614_][4-NO_2_-PhO]	[96]
PESU	30.2^b^	308	354.638	1. Good mechanical properties 2. Good chemical resistance 3. Can be used for water purification	1. Low CO_2_ permeability 2. Rapid physical aging 3. Relatively average CO_2_/N_2_ selectivity	1. [E_3_Py] [NTf_2_] 2. [aemmim][Tau] 3. [N_11_][Gly]+MDEA 4. [Emim][MDEGSO4] 5. [C_4_Py][NTf_2_] 6. [E_3_Py] [NTf_2_]	[28]
TPESU	35.5^b^	308	354.638	1. Good mechanical properties 2. Good chemical resistance 3. Can be used in water purification	Relatively average CO_2_/N_2_ selectivity	1. [E_3_Py] [NTf_2_] 2. [aemmim][Tau] 3. [N_11_][Gly]+MDEA 4. [Emim][MDEGSO_4_]	[28]
bh-MgO	1.76^a^	308	-	1. Thermal stability of up to 583 K 2. High CO_2_ permeability at 10–30% CO_2_ loading capacity 3. High CO_2_/N_2_ selectivity from 0-30% CO_2_ loading capacity	Relatively low CO_2_ permeability at CO_2_ 0–5% loading capacity	1. [C_4_Py][NTf_2_] 2. [E_3_Py] [NTf_2_]	[97]
Pebax/PBE	48.2^b^	308	100	1. Good thermal property (thermally stable up to 543K) 2. Good mechanical property	Thermal degradation at temperatures above 543 K	1. [E_3_Py] [NTf2] 2. [aemmim][Tau] 3. [N_11_][Gly]+MDEA 4. [Emim][MDEGSO_4_]	[98]
PSF-NH2-MIL-125(Ti)	29.2^b^	303	300	1. Relatively high CO_2_ permeability (9.5) 2. Good pressure-resistant property	1 Relatively average CO_2_/N_2_ selectivity at 30% CO_2_ loading capacity 2. Increase in pressure, at constant temperature, leads to a decrease in membrane permeability with respect to CO_2_	1. [E_3_Py] [NTf_2_] 2. [aemmim][Tau] 3. [N_11_][Gly]+MDEA 4. [Emim][MDEGSO_4_]	[99]
Matrimid/MIL-53	51.8^b^	308	300	1. Good permeability 2. Good selectivity 3. Good thermal resistance	At 20 wt.% MOF loading, selectivity decreased significantly	1. [E_3_Py] [NTf_2_] 2. [aemmim][Tau] 3. [N_11_][Gly]+MDEA 4. [Emim][MDEGSO_4_]	[100]
PVC-g-POEM/H_ZIF-8	13.7^b^	308	-	1. Good mechanical stability 2. High gas permeation	-	1. [E_3_Py] [NTf_2_] 2. [aemmim][Tau] 3. [N_11_][Gly]+MDEA 4. [Emim][MDEGSO_4_] 5. [C_4_Py][NTf_2_] 6. [E_3_Py] [NTf_2_]	[101]
NH_2_-MIL-125(Ti)	50^b^	308	900	1. High permeation of gases 2. High CO_2_/N_2_ selectivity	Decrease in permeability with a relative increase in selectivity.	1. [E_3_Py] [NTf_2_] 2. [aemmim][Tau] 3. [N_11_][Gly]+MDEA 4. [Emim][MDEGSO_4_] 5. [C_4_Py][NTf_2_] 6. [E_3_Py] [NTf_2_]	[101]
UiO-66-NH2-PEBA	72^b^	293	10.1325	1. High permeability 2. High CO_2_/N_2_ selectivity 3. Increase in permeability at increased temperature 4. Good chemical stability	Decrease in selectivity with an increase in temperature	1. [C2OHmim][Gly] 2. [BMIM][PF_6_]-MEA 3. [EMIM] [BF_4_]-MEA 4. [P_66614_][4-NO_2_-PhO]	[102]
MOF@COF/PSf-5	46.7^b^	298	100	48% Increase in permeability with the addition of 5 wt% MOF@COF fillers	15% reduction in CO_2_/CH_4_ selectivity from 26.1 to 22.1 due to the addition of 5 wt% MOF@COF fillers	1. [C2OHmim][Gly] 2. [BMIM][PF_6_]-MEA 3. [EMIM] [BF_4_]-MEA 4. [P_66614_][4-NO_2_-PhO]	[102, 103]
ZIF-8/6FDA-durene diamine	17.0^b^	673	200	1. High Permeability. 2. Increase in the permeation of N_2_, O_2_, CH_4_, and CO_2_ for an in increase in ZIF-8 loading in the mixed matrix membrane 3. High thermal resistance property (up to 769 K).	Decrease in the selectivity for gas pairs CO_2_/N_2_ and CO_2_/CH_4_ for an increase in ZIF-8 loading in the mixed matrix membrane	1. [E_3_Py] [NTf_2_] 2. [aemmim][Tau] 3. [N_11_][Gly]+MDEA 4. [Emim][MDEGSO4] 5. [C_4_Py][NTf_2_] 6. [E_3_Py] [NTf_2_]	[104]
Sod-ZMOF/Matrimid	43.4^b^	308	400	1. High thermal stability 2. High chemical stability 3. High mechanical stability	Thermal degradation at temperatures above 587 K.	1. [E_3_Py] [NTf_2_] 2. [aemmim][Tau] 3. [N_11_][Gly]+MDEA 4. [Emim][MDEGSO_4_] 5. [C_4_Py][NTf_2_] 6. [E_3_Py] [NTf_2_]	[105]
**Ionic Liquids**							
**Type of Ionic liquid**	**Carbon capture efficiency (%/**$\frac{mol\;{CO}_{2}}{mole\;IL}$**) (selectivity (-)**	**Conditions application**	**for**	**Strengths**	**Weaknesses**	**Selected Blends**	**References**
		**T(K)**	**P(kPa)**				
[bmim][PF_6_]	0.75^a^	298	8000	1. Stable in the presence of oxygen or water 2. Low viscosity	Thermal degradation at temperatures above 373 K	1. PVAm/PSf HF 2. PVAm/PSf HM 3. PVAm/PPO 4. polyvinylamine (PVAm)/piperazine glycinate (PG) 5. hydrophilic polymeric membrane based on microfibrillated cellulose (MFC) 6. bh-MgO 7. UiO-66-NH2-PEBA 8. DA-PEI-TiO2 9. ZIF-11/Pebax®2533 10. [E_3_Py] [NTf_2_] 11. [C_4_Py][NTf_2_]	[106]
[P_66614_][4-Me-PhO]	0.91^a^	303	-	1. Excellent absorptive capacity 2. Good CO_2_ absorption enthalpy 3. High thermal stability	-	1. PVAm/PSf HF 2. PVAm/PSf HM 3. PVAm/PPO 4. polyvinylamine (PVAm)/piperazine glycinate (PG) 5. hydrophilic polymeric membrane based on microfibrillated cellulose (MFC) 6. bh-MgO 7. UiO-66-NH_2_-PEBA 8. DA-PEI-TiO2	[107]
[P_66614_][4-Cl-PhO]	0.82^a^	303	-	1. Excellent adsorption capacity 2. Good adsorption enthalpy 3. High thermal stability	Thermal degradation at temperatures above 550 K	1. PVAm/PSf HF 2. PVAm/PSf HM 3. PVAm/PPO 4. polyvinylamine (PVAm)/piperazine glycinate (PG) 5. hydrophilic polymeric membrane based on microfibrillated cellulose (MFC) 6. bh-MgO 7. UiO-66-NH_2_-PEBA 8. DA-PEI-TiO_2_	[107]
[P_66614_][4-NO_2_-PhO]	0.30^a^	303	-	1. Good adsorption enthalpy 2. High thermal stability.	Thermal degradation at temperatures above 492 K	1. PVAm/PSf HF 2. PVAm/PSf HM 3. PVAm/PPO 4. polyvinylamine (PVAm)/piperazine glycinate (PG) 5. hydrophilic polymeric membrane based on microfibrillated cellulose (MFC) 6. bh-MgO 7. UiO-66-NH_2_-PEBA 8. DA-PEI-TiO_2_ 9. ZIF-11/Pebax®2533 10. [E3Py] [NTf2] 11. [C4Py][NTf2]	[107]
[emim][Tf_2_N]	0.6^a^	333.15	6000	1. Good CO_2_ absorption 2. High thermal stability	Thermal degradation at temperatures above 450 K	1. ZIF-11/Pebax®2533 2. [E3Py] [NTf2] 3. [C_4_Py][NTf_2_]	[108]
[aemmim][Tau]	0.9^a^	323.15	-	1. little heat is required for regeneration 2. Good thermal stability	Thermal degradation at temperatures above 323.15 K	1. ZIF-11/Pebax®2533 2. [E_3_Py] [NTf_2_] 3. [C_4_Py][NTf_2_]	[109]
[BMIM][BF_4_]-MEAY	0.72^a^	298	600	1. High absorption capacity 2. High chemical stability 3. Low viscosity 4. High recyclability	-	1. UiO-66-NH_2_-PEBA 2. DA-PEI-TiO_2_ 3. PVAm/PSf HF 4. PVAm/PSf HM 5. PVAm/PPO	[110]
[EMIM] [BF_4_]-MEA	0.6^a^	298	500	1. High absorption capacity 2. Good chemical stability	-	1. UiO-66-NH_2_-PEBA 2. DA-PEI-TiO_2_ 3. PVAm/PSf HF 4. PVAm/PSf HM 5. PVAm/PPO	[110]
[BMIM][PF_6_]-MEA	0.525^a^	298	550	1. Good absorption capacity. 2. Good chemical stability	-	1. PVAm/PSf HF 2. PVAm/PSf HM 3. PVAm/PPO 4. polyvinylamine (PVAm)/piperazine glycinate (PG) 5. hydrophilic polymeric membrane based on microfibrillated cellulose (MFC) 6. bh-MgO 7. UiO-66-NH_2_-PEBA 8. DA-PEI-TiO_2_	[110]
[C_2_OHmim][Gly]	0.575^a^	303.15	10	1. High thermal stability 2. High recyclability at temperatures between 373 and 393 K 3. Good chemical stability	Thermal degradation at temperatures above 523 K	1. PVAm/PSf HF 2. PVAm/PSf HM 3. PVAm/PPO 4. polyvinylamine (PVAm)/piperazine glycinate (PG) 5. hydrophilic polymeric membrane based on microfibrillated cellulose (MFC) 6. bh-MgO 7. UiO-66-NH_2_-PEBA 8. DA-PEI-TiO_2_	[111]
[EOMmim][PF_6_]	2.8606^a^	298	4656	1. High thermal stability 2. High chemical stability 3. Low viscosity	Decrease in Solubility with an increase in temperature	1. PVAm/PSf HF 2. PVAm/PSf HM 3. PVAm/PPO 4. polyvinylamine (PVAm)/piperazine glycinate (PG) 5. hydrophilic polymeric membrane based on microfibrillated cellulose (MFC) 6. bh-MgO 7. UiO-66-NH_2_-PEBA 8. DA-PEI-TiO_2_	[112]
[EOMmim][Tf_2_N]	2.0^a^	303.15	4500	1. High thermal stability 2. High chemical stability 3. Low viscosity	Decrease in solubility at increased temperature	1. ZIF-11/Pebax®2533 2. [E_3_Py] [NTf_2_] 3. [C_4_Py][NTf_2_]	[112]
[Emim][MDEGSO_4_]	1.25^a^	303.15	4500	1. High thermal stability 2. High chemical stability 3. Low viscosity	Decrease in solubility for an increase in temperature	1. ZIF-11/Pebax®2533 2. [E_3_Py] [NTf_2_] 3. [C_4_Py][NTf_2_]	[112]

Hint: ^a^ = CO_2_ adsorptive capacity of the ionic liquid/membrane; ^b^ = selectivity.

**Table 4:** Selectivities and permeabilities of various membranes.

Type of membrane/Nomenclature	Selectivity	Permeability (m^3^(STP)/(m^2^ bar h)/GPU/Barrer)	Ref
m-DAC	29.8	-	[92]
PVAm/PSf HF	230	0.022^a^	[93]
PVAm/PSf HM	133	-	[93]
PVAm/PPO	68	2.3^a^	[93]
Pebax/Zeolite Y	30	940^b^	[94]
polyvinylamine (PVAm)/piperazine glycinate (PG)	143	1100^b^	[95]
hydrophilic polymeric membrane based on microfibrillated cellulose (MFC)	500	350	[96]
PESU	30.2	-	[28]
TPESU	35.5	-	[28]
bh-MgO	-	179.2^c^	[97]
Pebax/PBE	48.2	-	[98]
PSF-NH_2_-MIL-125(Ti)	29.2	-	[99]
Matrimid/MIL-53	51.8	-	[100]

Hint: ^a^ = m^3^(STP)/(m^2^ bar h); ^b^ = GPU; ^c^ = Barrer.

There are several factors that affect membrane-IL performance in the process of capturing CO_2_ from a post-combustion operation. These factors can be enhanced to improve the performance of the hybrid system. Several of these factors include:

• Pore diameter/size: The size of the pores of the hybrid membrane-IL material should be such that it can selectively accommodate carbon molecules. The diameter of the pores should suit that of the carbon molecules.
• Selectivity: Selectivity is the property of the membrane-IL that gives it the ability to selectively adsorb carbon molecules, while it rejects the entry of other component materials. The membrane-ILs are designed to be task specific, and as such, do not adsorb more than the required component.
• Longevity: This property, alongside the regeneration characteristic of the ILMs, determines how many times the ILMs can be used and reused.

Cellulose acetate (CA) is regarded as the most popular membrane material utilized for water applications due to its inherent qualities, low cost, amazing potential flow, extended lifespan, low maintenance requirements, minimal membrane fouling and high hydrophilicity [113]. In recent years, interests in the use of hybrid (organic-inorganic) membranes in operations such as reverse osmosis (RO), nanofiltration, and ultrafiltration has increased [114]. RO is the most common operation which is mostly employed when cellulose diacetate (CA), cellulose triacetate, or a combination of both are used [115]. The most widely used natural polymer (cellulose) is converted into CA membranes by an acetylation procedure. CA membranes provide the following benefits: neutral surface, good resistance to free chlorine (at low level), very hydrophilic surface, and high potential water flux. They are also reasonably inexpensive. However, there are numerous drawbacks of CA membranes, including their limited pH operating range, vulnerability to bacterial attack, can be compressed under high pressure, and suitable for application under limited temperature range. In some major desalination facilities, the growth of bacteria on the surfaces of the membrane-forming biological filters is a significant issue that is very challenging to eradicate, either through disinfection or chemical cleaning. Extra-cellular polymeric substances (EPS), which are layers that provide protection against biocides for bacteria, are a type of polymer that microorganisms can exert to create biofilms with sturdy structures. By removing organic and inorganic elements from the environment around it, the microorganisms in the biofilm can obtain nutrition and continue to exist [115]. Further investigation of a system involving the creation of a hybrid cellulose acetate membrane employed in reverse osmosis was conducted. At a desalination rate of 35 g L^− 1^ and pressure of 60 bar, the salt rejection was 94 %. El-Din et al. [114] described a composite cellulose acetate membrane which exhibited a desalination rate of 5 g L^− 1^ within an operating pressure of 35 bar, with an estimated salt rejection of 96 %. Moreover, the forward osmosis (FO) method involves the exploitation of an osmotic pressure gradient as the driving force, thus making the membrane system energy-efficient. This is in contrast to applied pressure driven membrane processes like nanofiltration (NF) and reverse osmosis (RO), which require intensive energy consumption. In a FO process, water moves voluntarily from a location of higher water chemical potential (feed solution) to a region of lower water chemical potential (drawn solution) over a selectively permeable membrane [116]. Despite recent improvements in FO, there are still a number of obstacles to be overcome before FO procedures may be widely used successfully. There is substantive internal concentration polarization (ICP) in almost all FO membranes. This is a significant obstacle to the application of FO and is thought to be the primary cause of the process's significant lower water flux than anticipated. ICP may be able to reduce water flux by more than 80% according to early FO investigations. ICP alongside supporting layer thickness and porosity are tightly connected [116]. Severe ICP is less likely in a thin membrane with a highly porous layer-support. A small layer-support however, results in a membrane with lower mechanical strength. Additionally, some of the advantages of FO that have been historically compiled may run into new difficulties. The investigated cellulose acetate membrane composite system was adopted in a forward osmosis procedure. The feed solution contained 0.2 mol L^−1^ NaCl and the drawn solution had 1.5 mol L^−1^ glucose. The salt rejection was recorded as 96.03 %.

Haemodialysis is a crucial medical technique for people with renal diseases. During administration, the patient's blood is purified using haemodialysis equipment (an artificial kidney) so as to eliminate uremic wastes, and the filtered blood is then returned to the patient's body. Thus, the integration of a cellulose acetate (CA) membrane system is crucial for the haemodialysis machine. Its job is to convey excess water and toxic wastes from the patient via metabolic activities through diffusive and convective phenomena [117]. The hydrophilic –OH moieties and acetyl groups on the polymer backbone cause the CA to swell. Superior transport properties, low protein adsorption, great water affinity, mechanical strength, outstanding film-forming ability, and high hydrophilicity are further features that set CA membranes apart. Since CA has these properties by nature, it is preferred in a variety of biomedical applications including controlled release or blood purification in chronic renal dysfunction – haemodialysis. The first generation of polymers employed in dialyzers were cellulose and its derivatives. The purpose of the polymeric haemodialysis membrane is to eliminate blood toxins by convection and diffusion. These materials are affordable, can be processed easily, are renewable, and have the potential for recycling. They also have huge volume applications. The only negative aspect of CA is that it is not biocompatible. This issue can be solved by adding a biocompatible material with good hydrophilicity to CA, which will increase the effectiveness of the dialysis enforced through the membranes [117]. The authors described a complex cellulose acetate system for the hemodialysis process. The bovine serum albumin rejection was revealed to be >90 %. Dumitriu et al. [118] described the hybrid cellulose acetate system for the hemodialysis to have a growth inhibition for antimicrobial assay of 30 %.

One of the most promising strategies for the effective treatment of industrial wastewater containing harmful heavy metal ions is nanofiltration (NF) combined with adsorptive membrane technologies [118, 119]. The surface of the nanofiltration (NF) membrane has many charges and nanoscale pores, thus making it a membrane separation method that sits between reverse osmosis (RO) and ultrafiltration (UF). Through pore sieving and the Donna effect, this surface charge plays a key role in the effective removal of heavy metal ions from wastewater. However, the efficacy of conventional NF polymer membranes is continuously declining since they are easily contaminated with heavy metals during wastewater treatment. Additionally, it is difficult to overcome the trade-off between membrane permeability and selectivity in such membranes [118]. Yang et al. [119] synthesized a hybrid cellulose acetate membrane system comprising of nanoparticles for the purpose of nanofiltration. The membrane was found to achieve maximum metal removal rates of 63.2, 64.1 and 70.2 % for Cu^2+^, Cd^2+^ and Cr^6+^, respectively.

Membrane fouling is a significant problem in pressure-driven membrane processes, and it has a huge impact on how they can be technologically and economically implemented on a large-scale. Membrane fouling can assume different forms ranging from biofouling to inorganic, colloidal, and organic fouling. In nanofiltration, biofouling, which is known to account for more than 45% of all membrane fouling is a significant problem.

The use of nanomaterials to improve the performance of conventional membranes is currently the subject of active research, and given the numerous industrial applications for NF, the relationship between nanotechnology and membrane processes should be assessed by taking performance improvements like increased water permeability and solute selectivity into account. Beisl et al. [120] synthesized a hybrid cellulose acetate membrane for the nanofiltration of wastewater. The doping of silver nanoparticles in the composite membrane revealed salt rejections of 96.4, 97.4, 83.5, and 90.4 % for MgSO_4_, NaCl, MgCl_2_ and Na_2_SO_4_, respectively. The doping of silver ions exchanged for zeolite in the membrane revealed salt rejections of 92.5, 93.3, 82.8, and 86.5 % for MgSO_4_, NaCl, MgCl_2_ and Na_2_SO_4_, respectively.

One of the most often utilized materials for creating ultrafiltration membranes is cellulose acetate (CA). Numerous researchers have created cellulose acetate membranes and assessed their compaction, as well as their hydraulic and osmotic permeability characteristics. Owing to benefits such as moderate flow, strong salt rejection qualities, cost effectiveness, ease of fabrication, and non-toxicity, cellulose acetate and its derivatives are suitable raw materials for membrane preparation to be used in ultrafiltration processes. It has recently been demonstrated that CA's thermal properties are enhanced by the addition of borates and phosphates. Typically, additives are added to polymers to enhance their qualities, thus, allowing for a larger range of applications for CA [121]. The authors described a cellulose acetate membrane composite for the ultrafiltration/purification of wastewater. The rejection percentages for pepsin, egg albumin and bovine serum albumin (BSA) were 42.5, 52.6, and 66.7 %, respectively. Mahendran et al. [122] synthesized high-performance hybrid cellulose acetate membranes for ultrafiltration. The results revealed a molecular weight cut-off of 69 kDa. Currently, ultrafiltration (UF) in particular and membrane separation methods have found sufficient application in the separation of solutes from molecular solutions.

The fundamental driver behind the development of UF as an industrial process is the advancement in polymer manipulation achieved by polymer blending [123]. Thus, CA also has limitations, including a very small temperature limit (max. 30 °C), small pH range (limited to pH 2–8), low chlorine resistance and increased compaction phenomenon which in turn shortens the membrane-life along with its inherent biodegradability which limits its extensive utilization. Therefore, membrane-blends based on desirable polymer-properties will help overcome the aforementioned shortcomings of homopolymeric membranes. The authors synthesized hybrid high-performance membranes for ultrafiltration of wastewater. When the membrane was doped with PEG, the cadmium ion/PEI (polyethyleneimine) complex ion rejection was estimated as 80.3%, whereas, when the membrane was doped with PVP, cadmium ion/PEI (polyethyleneimine) complex ion rejection was revealed to be 76.4 %.

Cellulose acetate membranes have also found use in gas separation processes. The membranes are synthesized using solvents such as dimethylformamide (DMF), N-methyl-2-pyrrolidone (NMP), acetone etc., which help to promote the selective trapping of CO_2_ due to the individual solvents’ abilities to capture carbon. Some examples of cellulose acetate membranes synthesized for selective carbon capture are presented in Table 5.

**Table 5:** Cellulose acetate membranes synthesized for carbon capture.

Type of membrane/Nomenclature	Filler/dopant	Permeability (Barrer/GPU)	Weaknesses	References
CA	ZIF-62 glass nanoparticles	84.8	• The increased loading of ZIF-62 from 8-12 wt.% resulted in an increase in density from 1.01-1.15 g/cm^3^ while the fractional free volume (FFV) decreased from 0.34 to 0.27. • Further increment in ZIF-62 loading from 8-10 wt.% in the polymer matrix resulted in a dramatic drop in CO_2_ permeability and CO_2_/CH_4_ selectivity from 84.8-55.5 Barrer and 35.3–26.2, respectively.	[124]
CA	-	45.58	• A thicker skin slows down the solvent–coagulant exchange and thus reduced the membrane's porosity. • With time, the permeability for CO_2_ and CH_4_ decreased with increased solvent evaporation.	[125]
CA	Silica nanocomposite membranes.	7.3	• The permeabilities of O_2_ and N_2_ gases decreased from 0.95 and 0.186 barrer to 0.489 and 0.092 barrer in the nanocomposite membrane. • There was a decrease in CO_2_ diffusivity with an increase in silica wt.% of the membrane.	[126]
CA	NH_2_-MIL-53(Al)	20.2	• Gas permeation gradually reduced while ideal selectivities were improved.	[127]
CA	NH_2_-MIL 53(Al)	3.82	• CO_2_ permeation decreased at increased feed pressures and at a fixed temperature of 40 °C.	[128]

## 5 Socio-economic impacts of membrane-ionic liquid systems

Despite the number of researches that were dedicated to synthesizing cellulose acetate membranes for CO_2_ capture, not enough of the information available has been able to fully address the issues associated with high carbon capture efficiencies. Hence, this paper hopes to explore the various possibilities of hybridizing ionic liquids with cellulose acetate membranes in order to increase their CO_2_ capture efficiencies. Merging membranes and ionic liquids, as stated earlier, helps to improve on the strengths of the individual materials and thus, addresses the weaknesses of the individual materials. However, these ionic liquids and membranes are usually very expensive which makes it difficult for large scale adoption of the process. The economic implication of some membranes and ionic liquids are as given in Table 6:

**Table 6:** Cost implication of some technically tested membranes and ionic liquids.

Membranes	Stability Tests	Component materials/Cost per gallon or per kg (euros)	Total Cost (euros)	Ref.
m-DAC	-	-	-	[92]
PVAm/PPO	• Excellent chemical stability • Low thermal stability	• Polyvinylamine (48.10) • Poly(phenylene oxide) (PPO) (46.40) • Methanol (193.0) • Formamide (70.90) • Ethylene glycol (57.23)	415.63	[93]
PVAm/PSf HM	• Low thermal stability	• Polyvinylamine (48.10) • Homemade (PSF HM) • Methanol (193) • Formamide (70.90) • Ethylene glycol (57.23)	369.23	[93]
PVAm/PSf HF	• Low thermal stability	• Polyvinylamine (48.10) • Polysulfone hollow fibers (23.33) • Methanol (193) • Formamide (70.90) • Ethylene glycol (57.23)	392.56	[93]
Pebax /Zeolite Y	• Humidity tests revealed excellent chemical stability • X-ray diffraction (XRD) analysis showed good mechanical stability	• Poly(ethylene glycol) (PEG200) (117.65) • Poly(ethylene glycol) dimethyl ether (PEG-DME500) (233.27) • Heptane (99%) (5.07) • Isopropanol (IPA, 99.9%) (5.07) • Ludox HS-30 colloidal silica (sio2, 30%) (5.07) • Aluminum isopropoxide (Al(O–CH(CH_3_)_2_)_3_, 98%) (7.10) • Tetramethylammonium bromide ((CH_3_CH_2_CH_2_)4N(2Br), 98%) (121.90) • Ethanol (99.5%) (2.03) •Pebax® 1657 (30.49) • Catalyst (Wacker® Catalyst OL) (16.26) • PDMS (Dehesive® 944) (40.60)	584.51	[94]
polyvinylamine (PVAm)/piperazine glycinate (PG)	• Scanning electron microscopy (SEM) and Fourier transform infrared (FTIR) results showed good mechanical and chemical stability	• Glycine (98.5%) (1.53) • Piperazine (99%) (31.54) • N-vinylformamide (NVF, 98%) (144.0) • Α,α′-azodiisobutyramidine dihydrochloride (AIBA, 97%) (59.0) • Sodium dodecyl sulfate (SDS, 99%) (71.0) • Isopropanol (IPA, 99.9%) (68) • Hydrochloric acid (hcl, Certificated ACS Plus) (28) • Ethanol (99.5%) (64) • Strong base anion-exchange resin (Purolites A600OH) (100) • *Biomax polyethersulfone* ultrafiltration substrate (Biomax PES) (396.0)	963.07	[95]
hydrophilic polymeric microfibrillated cellulose (MFC) membrane	• Scanning electron microscopy (SEM) and Fourier transform infrared (FTIR) results showed good mechanical and chemical stability	• Microfibrillated cellulose (MFC) (85) •Birch bleached kraft pulps (393) • Polyvinylamine (Lupamin® 9095) (48.10)	526.1	[96]
bh-MgO	-	• Magnesium acetate tetrahydrate ((CH_3_COO)_2_Mg∙ 4H2O, 98%) (56) • Oxalic acid (HO_2_CCO_2_H, 98%) (202) • 4- styrenesulfonic acid sodium salt hydrate (H_2_C = CHC_6_H_4_SO_3_Na·xh2o) (148.0) • Potassium persulfate (K_2_S_2_O_8_, 99%) (46.10) • Ethanol (C_2_H_5_OH, 99.5%) (28) • PVC-g-POEM graft copolymer Poly(vinyl chloride) (PVC, average Mw of ∼233,000, average Mn of ∼99,000) (146) • Poly(oxy-ethylene methacrylate) (POEM, poly(ethylene glycol)methyl ether methacrylate, Mn ∼ 475 g/mol) (86.10) • 1, 1, 4, 7, 10, 10-hexamethyltriethylenetetramine (HMTETA, 99%) (150) • Copper (I) chloride (cucl, 99%) (1, 030) • N-Methylpyrrolidone (NMP) • (105) • Tetrahydrofuran (THF) (82.30) • Methanol (193.0)	2274.5	[97]
[bmim][PF_6_]	• Stable in the presence of oxygen or water • Low thermal stability	• 1-butylbromide (66.08) • 1-methylimidazole (36) • Sodium tetrafluoroborate (49.10) • Methylene dichloride (57.30) • Potassium hexafluorophosphate (47)	255.48	[106]
[P_66614_][4-Me-PhO]	• High thermal stability	• Phosphonium hydroxide (309) • Phenol (37.60)	346.6	[107]
[P_66614_][4-Cl-PhO]	• High thermal stability	• Phosphonium hydroxide (309) • Phenol (37.60) • Chlorine (1, 490)	1836.6	[107]
[P_66614_][4-NO_2_-PhO]	• High thermal stability	• Phosphonium hydroxide (309) • Phenol (37.60) • Nitrogen oxide (240)	586.6	[107]
[emim][Tf_2_N]	• High thermal stability.	• Trifluoromethylsulfonyl (80.40) • 1-ethyl-3-methylimidazolium (147)	227.4	[108]
[aemmim][Tau]	• High chemical stability	• Sodium (NaOH) solution (28.60) • Methanol (193) • 1,2- dimethylimidazolium (67.70) • Acetonitrile (119) • 2-bromoethylamine hydrobromide (28)	436.3	[109]
[BMIM][BF_4_]-MEAY	• Good chemical stability	• N-methylimidazole (36) • Monoethanolamine (≥99.5%) (164) • Chlorobutane (≥98%) (31.60) • Ethyl bromide (≥98%) (61.70) • Sodium tetrafluoroborate (≥98%) (49.10) • Potassium hexafluorophosphate (≥98%) (47.00)	389.4	[110]
[EMIM] [BF_4_]-MEA	• Good chemical stability	• N-methylimidazole (36) • Monoethanolamine (≥99.5%) (164) • Chlorobutane (≥98%) (31.60) • Ethyl bromide (≥98%) (61.70) • Sodium tetrafluoroborate (≥98%) (49.10) • Potassium hexafluorophosphate (≥98%) (47.00)	389.4	[110]

As highlighted above, the costs of the desired materials are very high. Relative to third world countries, the costs may even rise further due to shipment and distribution prices that may discourage fast rising industries from purchasing them. Thus, there is need to search for viable alternatives as a means of curbing such issues. One innovative way by which that can be done is to use readily available materials, such as cellulose acetate, as substitute for some expensive existing membrane materials/precursors. In addition, the incorporation of GO nanoparticles is also a viable option as it helps improve upon the mechanical stability and porosity of the membrane matrix. Graphene oxide powder can be synthesized from graphene which can be sourced from waste materials such as broken HB pencils. This can help improve the mechanical strength of the synthesized membrane.

Compared to the other systems highlighted in Table 7, graphene-oxide cellulose acetate system not only serves as a more cost-effective material for carbon capture, but it is considerably better when compared by virtue of its carbon retention. This is a viable proof/alternative approach for capturing carbon while still maintaining a high level of selectivity and carbon capture efficiency. Furthermore, for the discussed membrane gas separation process, the presence of gaseous impurities such as NOx/N_2_, O_2_, CH_4_, H_2_S and SO_2_ can contaminate the membrane-GO-ionic liquid system, however, this can be handled via gas pretreatment/desulfurization. Therefore, if it is so desired, some other techniques involving the use of SILMs that have the ability to strip CO_2_ and SO_2_ [129,130] may be adopted. The study by Zhang et al. [131] also informs the ability of SILMs containing (1-butyl-3-methylimidazolium tetra-fluoroborate and 1-butyl-3-methylimidazolium triflfluoromethanesulfonate) as ionic liquids which have the capacity for stripping H_2_S, CH_4_ and CO_2_ thus implying that some choice ionic liquids have wider potentials for multiple gas adsorption. Interfacially engineered SLMs have been produced via vapor cross-linking for enhanced selective separation of N_2_ and CO_2_ [132].

**Table 7:** Comparative data of the proposed ILM system versus other systems.

Type of system	CO_2_ retention	Ref
[P_66614_][4-NO_2_-PhO]	0.30	[107]
[emim][Tf_2_N]	0.6	[108]
[BMIM][BF_4_]-MEAY	0.72	[110]
[EMIM] [BF_4_]-MEA	0.6	[110]
[C_2_OHmim][Gly]	0.575	[111]
Membrane contactor and [emim][Ac]	0.45	[72]
Membrane contactor and 1-ethyl-3-methylimidazolium ethyl-sulfate	0.28	[66]
Graphene oxide-Cellulose acetate membrane	0.8	This work

## 6 Projecting a circular economy

As earlier stated, the various methods towards CO_2_ stripping and material utilization are important in the conservation of the earth. Regeneration of the spent adsorbent, reutilization and storage are important steps in curbing the concerns associated with carbon generated from industrial process plants. Figure 10, gives an ideal breakdown of the conversion process of CO_2_ generated from a power plant. The produced flue gas leaves the power plant, and carbon dioxide can be separated by adsorption using a sorbent/membrane and by absorption using a solvent. The CO_2_ captured can be used to produce urea, methanol, supercritical CO_2_, propylene as well as injected in oil wells for enhanced oil recovery.

**Figure 11:**
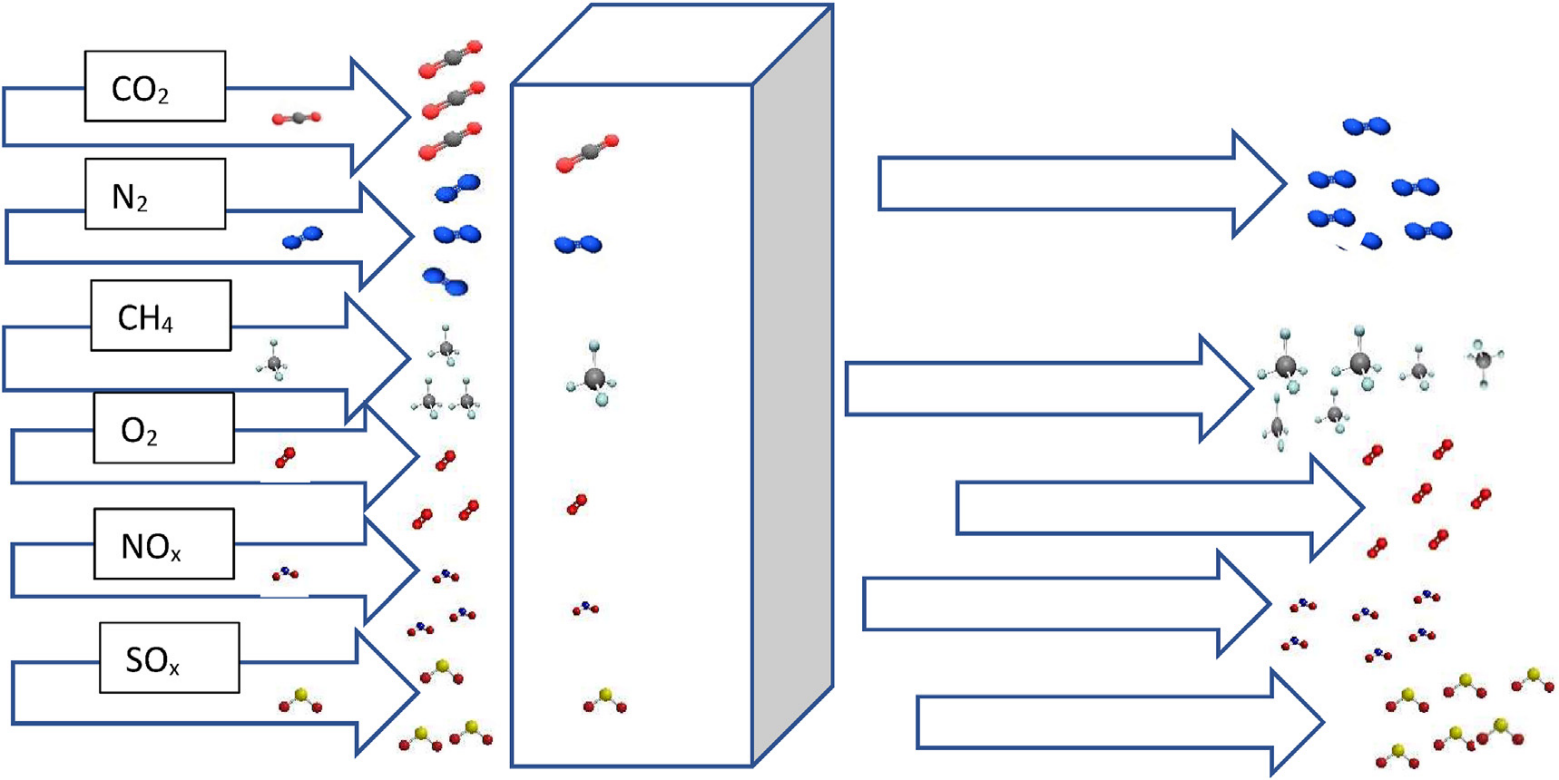
Mechanism for gas separation using a membrane.

The fundamental principle of membrane technology is that a permeable gas, after adsorbing unto the surface of the membrane material at a high pressure, diffuses through the membrane layer and desorbs as a permeate gas on the opposite side of the porous membrane. Nonpermeable gases are the gases that are left over at the high-pressure side of the membrane. Therefore, how efficiently CO_2_ adsorbs unto the membrane's surface and how well it diffuses on the other side of the membrane greatly influences how well CO_2_ can be separated [134, 135]. Figure 11, illustrates a mechanism for flue gas separation using a membrane. Flue gas generated from biogas/flue gas source passes through a membrane with various component gases (CO_2_, CH_4_, N_2_, NOx, SOx and O_2_) being separated.

## 7 Mechanism for CO_2_ adsorption/gas purification via membrane separation

Similar to the membrane-based post-combustion CO_2_ separation process, which requires a wide area membrane due to the low-pressure difference between the feed gas and permeate, more than half of the operation costs are incurred by the vacuum pump used to evacuate the permeate side of the membrane. The CO_2_ permeability across the membrane is therefore more significant than its CO_2_ selectivity for lowering the cost of the membrane module. However, because the feed gas stream used in pre-combustion gas separation, has a high pressure, neither a compressor nor a vacuum pump is needed. Therefore, having an effective CO_2_ separation process such as that (postcombustion CO_2_ capture) informed by this study requires both CO_2_ selectivity and CO_2_ permeability, with minimal operating [136]. For the cellulose acetate-IL-GO membrane composite that is CO_2_ selective, in a flue gas system comprising of other gases, CO_2_ is retained within the membrane pores while other gases permeate the membrane as presented in Figure 11. Table 8 shows some additional commonly used cationic and anionic IL-pairs that have some good measures of compatibility with the aforementioned CA-membrane recommended in this study.

**Table 8:** Commonly used cation and anion pairs of ILs in membranes for CO_2_ separation.

Type of ion	Cation	Structure	Name	Anion	Structure	Membrane Application	Ref.
Imidazolium	[C_3_H_5_N_2_] ^+^		Hexafluorophosphate	[PF_6_]^-^		Pebax1074/1-butyl-3- methylimidazolium hexafluorophosphate ([BMIM][PF6])	[59]
Phosphonium	[PH_4_] ^+^		Bis(trifluoromethanesulfonyl)imide	[Tf_2_N]^-^		PEG/tetradecyl(trihexyl)phosphonium bis(triflamide)	[137]
Pyridinium	[C_5_H_6_N] ^+^		Dicyanamide	[DCA] ^-^		Pyridinium containing amide based poly(ionic liquid)s(PAPILs)	[138]
Pyrrolidinium	[C_4_H_10_N] ^+^					Poly([pyr11][NTf_2_])–[pyr_14_][NTf_2_] composite membrane	[139]

## 8 Conclusion

This review article explored CO_2_ capture from flue/biogas sourced from biomass. Over the years, various techniques have been employed with a clear focus on membrane-ionic liquid systems as a means of controlling the high carbon content of the atmosphere. The different techniques for CO_2_ capture include: chemical absorption, membrane separation, chemical looping, etc. In lieu of the fact that these techniques on their own, have shown some measures of reliability in spite of the different technologies involved in capturing CO_2_, the dearth in mobilizing hybrid systems comprising of composite ionic liquid-membrane systems for carbon capture operations has propelled the content discussed herein. Several ionic liquids and membrane systems alongside their properties, nature and performances were discussed in relation to their CO_2_ capture potentials. This was done in order to bring to bear, the potentials that underlay the use of some poor, moderate, low and high performing ionic liquid systems as a way of ensuring property compensation which helps to boost CO_2_ retention in the hybrid systems. The investigation further explored the advantages of the aforementioned hybrid systems over traditional liquid absorbents such as monoethanolamine (MEA) solutions in removing CO_2_ in a post-combustion process. Some trendy ionic liquid-membrane materials were highlighted as somewhat suitable and efficient for capturing CO_2_. Their compensations for the inefficiencies in permeability and selectivity data of their relative counterparts, makes them suitable candidates for further investigation. Polymeric ionic liquids (PILs) have demonstrated enhanced solubility as a result of the evident property-modification of the IL by the polymer network, as well as sufficient permeation and viscosity when used with membranes. Cellulose acetate membranes and their use in various areas including water filtration, food processing and health, were considered. Gas separation techniques involving cellulose acetate membranes as well as the potential advancements in their synthesis with ionic liquids were explored. Low corrosivity, excellent thermal stability, insignificant vapor pressure, low thermal expansion, low cost, ease of synthesis, high CO_2_ absorption capacity, and selectivity are all advantages of PILs which makes them deserve further investigation for future application with membrane-systems for CO_2_ capture in a post-combustion process. Also, of the membrane-ionic liquid systems discussed, CA-methyl ammonium nitrate-GO membrane hybrid system is a potentially viable candidate with high CO_2_ capture of >80%; this is due to the improved porosity, thermal/mechanical stability, good selectivity and low CO_2_ permeability imposed by the combined constituents which help to boost its CO_2_ retention.

## Declarations

### Author contribution statement

Samuel Eshorame Sanni: Conceived and designed the experiments; Analyzed and interpreted the data; Contributed reagents, materials, analysis tools or data; Wrote the paper.

Denen A. Vershima: Performed the experiments; Wrote the paper.

Emeka Emmanuel Okoro; Babalola Aisosa Oni: Contributed reagents, materials, analysis tools or data.

### Funding statement

This research did not receive any specific grant from funding agencies in the public, commercial, or not-for-profit sectors.

### Data availability statement

Data included in article/supp. material/referenced in article.

### Declaration of interest's statement

The authors declare no competing interests.

### Additional information

No additional information is available for this paper.
